# Activation of TGR5 Ameliorates Streptozotocin-Induced Cognitive Impairment by Modulating Apoptosis, Neurogenesis, and Neuronal Firing

**DOI:** 10.1155/2022/3716609

**Published:** 2022-04-15

**Authors:** Ronghao Mu, Xian Wu, Danhua Yuan, Jiajia Zhao, Susu Tang, Hao Hong, Yan Long

**Affiliations:** ^1^Department of Pharmacology, Key Laboratory of Neuropsychiatric Diseases, China Pharmaceutical University, Nanjing 211198, China; ^2^School of Pharmacy, The Key Laboratory of Major Autoimmune Diseases, Anhui Medical University, Hefei 230032, China

## Abstract

Takeda G protein-coupled receptor 5 (TGR5) is the first known G protein-coupled receptor specific for bile acids and is recognized as a new and critical target for type 2 diabetes and metabolic syndrome. It is expressed in many brain regions associated with memory such as the hippocampus and frontal cortex. Here, we hypothesize that activation of TGR5 may ameliorate streptozotocin- (STZ-) induced cognitive impairment. The mouse model of cognitive impairment was established by a single intracerebroventricular (ICV) injection of STZ (3.0 mg/kg), and we found that TGR5 activation by its agonist INT-777 (1.5 or 3.0 *μ*g/mouse, ICV injection) ameliorated spatial memory impairment in the Morris water maze and Y-maze tests. Importantly, INT-777 reversed STZ-induced downregulation of TGR5 and glucose usage deficits. Our results further showed that INT-777 suppressed neuronal apoptosis and improved neurogenesis which were involved in tau phosphorylation and CREB-BDNF signaling. Moreover, INT-777 increased action potential firing of excitatory pyramidal neurons in the hippocampal CA3 and medial prefrontal cortex of ICV-STZ groups. Taken together, these findings reveal that activation of TGR5 has a neuroprotective effect against STZ-induced cognitive impairment by modulating apoptosis, neurogenesis, and neuronal firing in the brain and TGR5 might be a novel and potential target for Alzheimer's disease.

## 1. Introduction

Alzheimer's disease (AD), the most common form of dementia, is characterized by progressive cognitive decline [1]. Between 2000 and 2018, reported deaths from AD have increased 146.2% [2], and it has been estimated to rise to 115 million worldwide by 2050 [3]. However, the pathogenic factor and etiopathogenesis of AD still remain unclear. AD can be categorized into late-onset sporadic AD (SAD) and early-onset familial AD (FAD), and the majority of AD cases are SAD [4]. In AD, hypometabolism of glucose and deficits in energy are observed. Production of adenosine 5′-triphosphate (ATP) from glucose metabolism in SAD declines to 50% or more throughout the progression of the disease [5]. Streptozotocin (STZ), a glucosamine-nitrosourea compound, has been commonly used to induce type 2 diabetes and insulin resistance in animals due to its activity to damage the pancreatic *β* cells [6, 7]. Previous studies have shown that intracerebroventricular STZ (ICV-STZ) injection produces cognitive deficits in mice [8–10]. Compared with widely used transgenic 3xTg-AD animals, the AD model of ICV-STZ mouse shows the dysfunction of energy homeostasis and multiple other effects of SAD abnormalities which are not caused by overexpression of transgenes of amyloid-*β* precursor protein (APP), presenilin-1 (PS1), and tau [11].

One of the main features of AD is energy impairment in the brain. Hypometabolism caused by decreased glucose uptake is observed in specific areas of the AD-affected brain. Glucose is the sole source of energy for the brain, but ketone bodies can be used only as a last resort [12]. However, neurons in the brain cannot synthesize and store glucose, and a continuous supply of glucose is necessary for neuronal activities including apoptosis, neurogenesis, and neuronal firing [13]. Decreased transport of glucose and reduced glucose utilization in the brain occur during normal aging and may cause AD [14]. Importantly, regulated neuron death plays key roles in biological processes including the development and survival of the central nervous system (CNS). Previous studies have confirmed that the deregulation of neuronal apoptosis leads to neurodegenerative diseases such as AD [15]. Dopamine neuronal apoptosis results in memory and reward dysfunction in a model of AD [16], and therapeutic drugs of AD suppress neuronal apoptosis in the hippocampus [17]. In addition, hippocampal neurogenesis plays an important role in structural plasticity and network maintenance, and emerging evidence has indicated that altered neurogenesis in the hippocampus represents an early critical event in the course of AD [18]. Brain-derived neurotrophic factor (BDNF) is modified that the precursor form of BDNF (pro-BDNF) causes neuronal apoptosis in AD [19], while the mature form of BDNF (mBDNF) is always neuroprotective and antiapoptotic [20]. More and more studies have shown that neuronal firing in the brain plays critical roles in memory which is supported by intrinsic plasticity that downregulates neuronal firing rates [21], and NMDA receptors subserve persistent neuronal firing in the dorsolateral prefrontal cortex during working memory [22]. AD starts from pure cognitive impairments and gradually progresses into degeneration of specific brain circuits, while neural firing instability at early AD stages triggers a vicious cycle leading to homeostasis network collapse [23].

Takeda G protein-coupled receptor 5 (TGR5) is the first known G protein-coupled receptor specific for bile acids while its activation improves glucose tolerance and insulin sensitivity [24]. Recent studies have shown that TGR5 is also expressed in microglia, astrocytes, and neurons in the brain [25, 26]. TGR5 activation has been demonstrated to have neuroprotective effects on stroke, atherosclerosis, and ischemia/reperfusion animal models [27–30]. INT-777, a specific TGR5 agonist, can increase energy expenditure, improve insulin sensitivity, and decrease inflammation [31]. Furthermore, INT-777 activates TGR5 to exert a protective effect against apoptosis and oxidative stress [32, 33]. To date, little attention has been paid to the participation of TGR5 in cognitive impairment induced by dysfunction of energy homeostasis. The aims of the present study were to investigate the neuroprotective effects of TGR5 on cognitive impairment in STZ-induced mice and the possible underlying mechanism involved in apoptosis, neurogenesis, and neuronal firing.

## 2. Materials and Methods

### 2.1. Animals

Male SPF grade ICR and C57BL/6 mice (6–8 weeks old) provided by the Medical Center of Yangzhou University (Yangzhou, China) were housed under conditions of a temperature of 23 ± 2°C, a humidity of 55 ± 5%, and a 12 h/12 h cycle of illumination, during which animals were given access to food and water ad libitum. All methods were endorsed by the Institutional Animal Care and Use Committee (IACUC) of China Pharmaceutical University (Nanjing, China). The animal approval numbers for experiments of IACUC were SYXK: 2016-0011, 2021-0011.

### 2.2. Drugs and Reagents

INT-777 (6*α*-ethyl-23(S)-methylcholic acid) was purchased from Intercept Pharmaceuticals (NY, USA). STZ (2-deoxy-2-(3-(methyl-3-nitrosoureido)-D-glucopyranose), CPP ((RS)-3-(2-carboxypiperazin-4-yl)-propyl-1-phosphonic acid, NMDA receptor antagonist), and BrdU (5-bromo-2′-dexoyuridine) were purchased from Sigma Aldrich (St. Louis, MO, USA). NBQX (2,3-dioxo-6-nitro-1,2,3,4-tetrahydrobenzo[f] quinoxaline-7-sulfonamide disodium salt, AMPA receptor antagonist) and gabazine (6-imino-3-(4-methoxyphenyl)-1(6H)-pyridazinebutanoic acid hydrobromide, GABA_A_ receptor antagonist) were purchased from MedChemExpress (New Jersey, USA). Adeno-associated virus of AAV2/9-CaMKII*α*-mCherry was purchased from BrainVTA (Wuhan, China). Primary antibodies were purchased from several companies: anti-TGR5, anti-BrdU, anti-NeuN, anti-Tau (Ser202), and anti-Tau5 from Abcam (Cambridge, USA); anti-BDNF from Santa Cruz Biotechnology (Heidelberg, Germany); anti-caspase-3, anti-Bcl-2, anti-Bax, anti-CREB, and anti-pCREB from Cell Signaling Technology (Massachusetts, USA), and anti-*β*-actin from Bioworld Technology (Minneapolis, USA). Horseradish peroxidase-conjugated anti-rabbit or anti-mouse secondary antibodies were from Bioworld Technology (Minnesota, USA), while donkey anti-rabbit Alexa Fluor 594 secondary antibody was purchased from Abcam (Cambridge, USA). The ADP/ATP (adenosine-5′-diphosphate/adenosine 5′-triphosphate) ratio assay kit and bile acid assay kit were purchased from Abcam (Cambridge, USA), and the glucose assay kit was purchased from Solarbio Technology (Beijing, China). The primers for TGR5 mRNA (forward: 5′-GATAATGTGCTGTCCCCACC-3′; reverse: 5′-AGCTGACCCAGGTGAGGAAC-3′) and GAPDH mRNA (forward: 5′-ACATTGTTGCCATCAACGAC-3′; reverse: 5′-ACGCCAGTAGACTCCACGAC-3′) were provided by Sangon Biotech (Shanghai, China). In the supplementary materials, primary antibodies such as anti-GSK3*β*, anti-pGSK3*β*, anti-NF-*κ*B p65, anti-Histone H3, anti-TNF-*α*, and anti-IL-1*β* were purchased from Cell Signaling Technology (Massachusetts, USA), while anti-Iba1 was purchased from Wako (Osaka, Japan). The TUNEL apoptosis assay kit was purchased from Beyotime Biotechnology (Shanghai, China).

### 2.3. Drug Treatments

**Figure 1:**
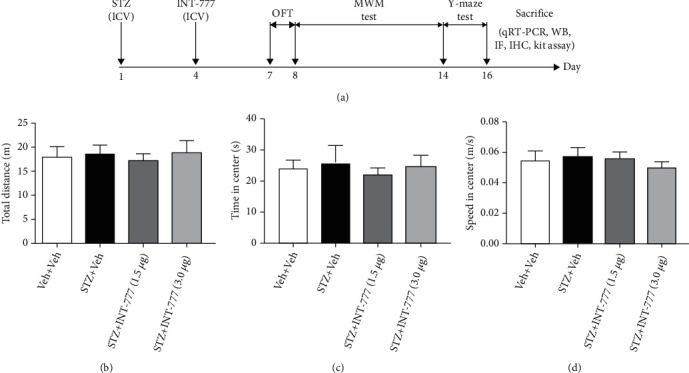
Effects of INT-777 on the locomotor activity in OFT in ICV-STZ mice. (a) Schematic illustrating the timeline for drug administration, behavioral tests, and biochemical analyses. ICR mice received INT-777 (1.5 or 3.0 *μ*g/mouse) or 0.1% DMSO after intracerebroventricular infusion of STZ or ACSF. (b–d) The OFT was evaluated for the following parameters: total distance moved (b), time spent in the center zone (c), and average speed moved (d). Values shown are expressed as the mean ± S.E.M.; *n* = 10 mice/group.

ICR mice were randomly assigned into four groups (*n* = 10/group): Veh+Veh, STZ+Veh, STZ+INT-777 (1.5 *μ*g/mouse), and STZ+INT-777 (3.0 *μ*g/mouse). STZ and INT-777 were dissolved in artificial cerebrospinal fluid (ACSF) containing 0.1% dimethyl sulfoxide (DMSO). After one-week adaption, the ICV-STZ mice were established by intracerebroventricular injection of a single dose of STZ (3 mg/kg) [9, 11, 34], while the control mice received the same volume (3 *μ*L) of ACSF. Three days later, INT-777 or ACSF containing 0.1% DMSO was infused into the same region of the brain. The dose of INT-777 was based on our previous study [35]. One week after the STZ injection, mice were subjected to a battery of behavioral tests and biochemical analyses (Figure 1(a)). The timeline for completion of the behavioral tests was based on previous studies [36, 37]. To label proliferating cells, ICR mice were intraperitoneally injected with BrdU (4 × 50 mg/kg at 2 h interval/d) for one day which was on the same day for STZ injection, and mice were killed 16 days after the final BrdU injection. To detect the changes of TGR mRNA and ADP/ATP ratio in the hippocampus and frontal cortex as well as glucose and bile acid in the serum, another separate set of groups of ICR mice (*n* = 8/group) were specially used for the kit assay.

C57BL/6 mice were randomly assigned into four groups (*n* = 8/group) and treated with the same administrations of STZ and INT-777. However, AAV2/9-CaMKII*α*-mCherry (300 nL) was bilaterally injected into the hippocampal CA3 and medial prefrontal cortex (mPFC) two weeks before STZ injection to mark excitatory pyramidal neurons in whole-cell patch-clamp recordings, and NBQX (5 *μ*M), CPP (5 *μ*M), and gabazine (10 *μ*M) were used in the bath solution of electrophysiological recordings in brain slices.

### 2.4. Stereotaxic Injection

Mice were first anesthetized with an intraperitoneal injection of 0.5% sodium pentobarbital (50 mg/kg) and then restrained onto a stereotaxic frame. STZ or ACSF was gradually infused with a volume of 3 *μ*L into the lateral ventricles (LV) of the brain at 1 *μ*L/min. The micropipettes were left in place for 5 min to minimize back flux of liquid. Mice were allowed to recover from anesthesia under a heating pad after surgery. Three days later, ACSF or INT-777 (1.5 or 3.0 *μ*g/3 *μ*L) was also infused into the LV. For C57BL/6 mice, AAV2/9-CaMKII*α*-mCherry (300 nL) was bilaterally injected into the mPFC or hippocampal CA3 two weeks before STZ injection to mark excitatory pyramidal neurons with red fluorescence. The injection coordinates (relative to bregma, mm) are the following: LV (AP: −0.3, ML: −1.0, and DV: −2.5), mPFC (AP: +2.0, ML: ±0.2, and DV: −2.5), and CA3 (AP: −1.7, ML: ±1.8, and DV: −2.0). One week after the stereotaxic injection of STZ, mice were subjected to behavioral tests.

### 2.5. Open Field Test (OFT)

Locomotor activity was evaluated in an open field chamber (a plastic box with dimensions of 50 cm × 50 cm × 40 cm divided into 144 squares), as previously described [38]. The mouse was placed in a corner square of the arena and allowed to freely explore the open field for 5 min. The total distance, the time spent in central squares, and the average speed were recorded by a video tracking system. The open field was cleaned with 75% ethyl alcohol and allowed to dry between tests.

### 2.6. Morris Water Maze (MWM) Test

Learning and memory of the animals were assessed in the MWM, as described previously [39, 40]. Each mouse was placed in a round pool (1.2 m diameter, 0.5 m height) filled with water to a 0.3 m depth at a temperature of 25°C. An escape platform with a diameter of 9 cm was placed at the midpoint of any quadrant of the pool, and visual cues were located around the room. The test consisted of five days of training period with visible and hidden platforms. The escape platform always stayed at a fixed position during the entire duration of training (5 days). On every trial, mice were put into the pool facing the pool wall. The visible platform training sessions were performed on days 1 and 2. During visible training trials, a small flag was affixed to the platform that was 1 cm below the surface of the water. Hidden platform training sessions in which the small flag was removed but the position of the platform remained the same were performed on days 3-5. There were four trials per day, each lasting 90 s, with an intertrial interval of 1 hour. Once on the platform, the trial ends after 10 s. If the mouse failed to find the platform after 90 s, it would be gently guided to the platform and left for 30 s before being removed. The time to find the platform (escape latency) was compared. On the final day (day 6), during the probe trial, mice were allowed to swim for 90 s while the platform is taken away. Escape latency, the time spent in the target quadrant, and the number of platform location crossings of mice were monitored by a camera above the center of the pool connected to Viewer 2 Tracking Software (Ji Liang Instruments, Shanghai, China).

### 2.7. Y-Maze Test

The Y-maze test was designed to assess short-term memory [41, 42]. The apparatus consists of three compartments (10 cm × 10 cm), which were connected with passages (4 cm × 5 cm). Each compartment was equipped with the same light. On day 1, each mouse was allowed to explore the compartment freely for 5 min. After 5 min, an electric shock (2 Hz, 125 ms, 10 V) was made available in two of the compartments while one compartment was left shock-free. The shock-free compartment was demarcated from the other two by a light. Each mouse was trained for 10 sessions, and each session was stopped once the mouse entered the shock-free compartment and stayed there for 30 s. If the mouse entered the shock-free compartment for the first time, it would be recorded as a correct choice. If the mouse did not enter this compartment, it would be guided to the compartment and allowed to stay for 30 s. On day 2 (testing trial), the mice were subjected to the test following the same procedures as on day 1 except the 5 min habituation period. The numbers of correct choices and the latency to make a correct choice were recorded manually.

### 2.8. Extraction of Total Protein and Western Blot (WB)

For extraction of total protein, tissues samples (*n* = 4/group) obtained from the hippocampus and frontal cortex were homogenized in ice-cold RIPA buffer, followed by centrifugation at 12000 g for 15 min. Protein concentrations in the supernatants were determined by the BCA protein assay kit (Beyotime Institute of Biotechnology, Shanghai, China).

A total of 40 *μ*g protein from each sample was separated electrophoretically in 8-12% sodium dodecyl sulfate polyacrylamide gel and transferred to polyvinylidene difluoride membranes. 5% nonfat milk was used to block the membranes for 1 hour at room temperature. Membranes were then incubated overnight at 4°C with respective primary antibodies for TGR5 (1 : 1000), Tau (Ser202) (1 : 5000), Tau5 (1 : 1000), BDNF (1 : 1000), CREB (1 : 1000), pCREB (1 : 1000), caspase-3 (1 : 1000), Bcl-2 (1 : 1000), Bax (1 : 500), and *β*-actin (1 : 3000), while for GSK3*β* (1 : 1000), pGSK3*β* (1 : 1000), NF-*κ*B p65 (1 : 1000), Histone H3 (1 : 1000), TNF-*α* (1 : 1000), and IL-1*β* (1 : 1000) in the supplementary materials.

Membranes were washed thoroughly with tris-buffered saline-tween 20 (TBST), then were incubated with horseradish peroxidase-conjugated secondary antibodies (1 : 3000) for 1 hour at room temperature. After washing, the bands were detected by enhanced chemiluminescence detection reagents and a gel imaging system (Tanon Science & Technology, Shanghai, China). Relative expression levels of proteins were normalized to *β*-actin or Histone H3.

### 2.9. Quantitative Real-Time Reverse Transcription PCR (qRT-PCR)

Tissue samples (*n* = 3/group) obtained from the hippocampus and frontal cortex were prepared. Using the RNA extraction kit and qRT-PCR kit (Sangon Biotech, Shanghai, China), we performed qRT-PCR according to the manufacturer's standard protocol. The threshold cycle (CT) was defined as the fractional cycle number at which fluorescence passed the fixed threshold. The relative levels of TGR5 mRNA were normalized to that of GAPDH and calculated using the *^ΔΔ^*CT method.

### 2.10. Immunofluorescence (IF) and Immunohistochemistry (IHC)

For tissue preparation, mice were anesthetized and transcardially perfused with 50 mL PBS (0.1 M) followed by 4% paraformaldehyde (PFA) in PBS. The brain was removed and subsequently submerged into 4% PFA overnight before being cryoprotected in 30% sucrose solution in PBS until they sank to the bottom of the container. Using a cryostat (CM3050S, Leica, Germany), the frozen brains were sectioned into 30 *μ*m coronal slices.

For IF, brain sections were washed in PBS three times (5 min each) and incubated with blocking solution (10% normal donkey serum, 1% BSA, and 0.3% Triton X-100 in PBS) for 1 h at room temperature. Sections were subsequently incubated with primary antibody in PBS overnight at 4°C. After washing in PBS three times (5 min each), sections were incubated with fluorescein-labeled secondary antibody in PBS for 1 h. After washing in PBS and staining with DAPI (Solarbio, Beijing, China), brain sections were mounted on positively charged slides with prolong anti-fade medium. Fluorescent images were taken using a fluorescence microscope (DM2000, Leica, Germany).

To stain TGR5 cells, rabbit anti-TGR5 (1 : 300) was used as the primary antibody while donkey anti-rabbit Alexa Fluor 594 (1 : 1000) was used as the secondary antibody. To label proliferating cells, brain sections were incubated with rat anti-BrdU (1 : 40) and mouse anti-NeuN (1 : 200) at 4°C overnight. After rinsing with PBS, the sections were incubated with Cy3-conjugated goat anti-rat IgG (1 : 100) for BrdU labeling and donkey anti-mouse IgG (1 : 100) for NeuN labeling.

For IHC in the supplementary materials, brain sections were heated on a water bath for 4 h in 0.3% Triton X-100 at 60°C followed by immersion in 3% H_2_O_2_ for 30 min and washed in PBS three times (5 min each). Sections were blocked with 5% BSA for 1 h and then incubated in anti-Iba1 (1 : 1000) primary antibody overnight at 4°C. On day 2, sections were washed in PBS three times (5 min each), incubated with biotinylated mouse anti-rabbit IgG at 37°C for 20 min, washed in PBS three times (5 min each), and incubated with streptavidin-biotin complex (SABC) at 37°C for 20 min. Diaminobenzidine (DAB) was used as the final chromogen at 37°C for the detection of target proteins. All images were taken in roughly the same imaging area, and the number of target cells was counted and quantified using NIH ImageJ software.

### 2.11. ADP/ATP Ratio Assay

Tissue samples (*n* = 3/group) obtained from the hippocampus and frontal cortex were prepared. Using the ADP/ATP ratio assay kit, we detected the ADP/ATP ratio according to the manufacturer's standard protocol. In brief, extraction of tissue samples and ATP reaction mix were first added and incubated for 2 min to analyze with a luminescence plate reader to measure ATP. After preparing ADP reaction mix and measuring luminescence levels again, ADP reaction mix was added to the same wells and incubated for 2 min to analyze with a luminescence plate reader to measure ADP.

### 2.12. Glucose Assay

Blood samples (*n* = 3/group) obtained from retroorbital venous plexus were prepared. Using the glucose assay kit, we detected the level of glucose in serum according to the manufacturer's standard protocol. In brief, serum samples and standards were first added and incubated for 15 min at 37°C. Then absorbance was analyzed at 505 nm by a spectrophotometer (UV-1900, Shimadzu, Japan).

### 2.13. Total Bile Acid Assay

Blood samples (*n* = 3/group) obtained from retroorbital venous plexus were prepared. Using the bile acid assay kit, we detected the level of total bile acid in serum according to the manufacturer's standard protocol. In brief, serum samples and standards were first added to wells. Then, probe mix was added to the same wells which were placed at 37°C for a 10 min incubation. Reaction mix was added, and absorbance was measured at 405 nm for 60 min at 37°C by a microplate reader (Multiskan FC, Thermo Scientific, USA).

### 2.14. Electrophysiological Recordings in Brain Slices

The methods of brain slice preparation and electrophysiological recordings were similar to those in previous studies [43–45]. In brief, after the full expressions of AAV, mice were anesthetized with isoflurane and the brains were rapidly removed and chilled in ice-cold sucrose solution containing (in mM) 40 NaCl, 4.5 KCl, 1.25 NaH_2_PO_4_, 25 NaHCO_3_, 148.5 sucrose, 10 glucose, 1 ascorbic acid, 3 Na pyruvate, 3 myo-inositol, 0.5 CaCl_2_, and 7 MgSO_4_, pH 7.3, 315 mOsm. Coronal brain slices (300 *μ*m) containing CA3 or mPFC were prepared in the same ice-cold sucrose solution using a vibratome (VT-1200s, Leica, Germany). Slices were then incubated in warm (32–34°C) sucrose solution for 30 min and transferred to ACSF composed of (in mM) 125 NaCl, 4.5 KCl, 1.25 NaH_2_PO_4_, 25 NaHCO_3_, 15 sucrose, 15 glucose, 2.5 CaCl_2_, and 1.3 MgSO_4_, pH 7.3, 315 mOsm, and allowed to cool to room temperature before electrophysiological recording. All solutions were continuously bubbled with 95% O_2_/5% CO_2_. Electrophysiological recordings were made using a MultiClamp700B amplifier and PClamp software (Molecular Devices, USA). The data were low-pass filtered at 2 kHz and digitized at 10 kHz with Digidata 1440 (Molecular Devices, USA). During recording, slices were submerged in normal, oxygenated ACSF and superfused (2 mL/min) at room temperature.

After expressions of AAV2/9-CaMKII*α*-mCherry, action potentials (APs) of mCherry-labeled excitatory pyramidal neurons in the CA3 or mPFC were recorded with intracellular depolarizing current injection (+70 pA) by a whole-cell current clamp. The pipette (3–5 M*Ω*) was pulled by a micropipette puller (P-97, Sutter Instrument) and filled with the internal solution (in mM: 105 K-gluconate, 5 NaCl, 10 HEPES, 2 MgATP, 0.5 NaGTP, and 0.2 EGTA, pH 7.3, 290 mOsm). Additionally, NMDA receptor antagonist CPP, AMPA receptor antagonist NBQX, and GABA_A_ receptor antagonist gabazine were added to ACSF solution for recording. Spike frequency of AP was analyzed with Clampfit 11.1 software.

### 2.15. TUNEL for Assessment of Neuronal Apoptosis

Coronal brain sections (30 *μ*m) were prepared as described above. Using the TUNEL apoptosis assay kit in the supplementary materials, we assessed neuronal apoptosis according to the manufacturer's standard protocol. DAPI nuclear staining was used to determine the total number of cells in a given area. TUNEL-positive cells were identified by the colocalization of both the TUNEL signal and DAPI.

### 2.16. Statistical Analysis

All experiments and data analyses were conducted blindly, including behavioral and biochemical analyses. Statistical analyses were performed using SPSS software (version 20.0; IBM, NY, USA), and graphs were generated by GraphPad Prism (version 7.0; GraphPad Software, CA, USA). All data are shown as the mean ± standard error of mean (SEM). Behavioral data of the Morris water maze were analyzed using two-way ANOVA followed by Dunnett's post hoc analysis. All other data were analyzed by one-way ANOVA followed by Dunnett's post hoc analysis for multiple comparisons. *P* < 0.05 was considered statistically significant.

## 3. Results

### 3.1. TGR5 Activation by INT-777 Has No Effect on Locomotor Activity in ICV-STZ Mice

To evaluate whether the INT-777 administrations contribute to the changes in locomotor activity in mice, the OFT was conducted. One-way ANOVA revealed that INT-777 treatments did not significantly alter the total distance (*F* [3, 36] = 0.14, *P* = 0.94; Figure 1(b)), the time in center (*F* [3, 36] = 0.33, *P* = 0.80; Figure 1(c)), and the average speed (*F* [3, 36] = 0.16, *P* = 0.92; Figure 1(d)). These results indicate that INT-777 effects on STZ-induced cognitive impairment are not due to nonspecific motor actions.

### 3.2. TGR5 Activation by INT-777 Ameliorates STZ-Induced Cognitive Deficits in Mice

Previous studies, including ours, have demonstrated impaired learning and memory in ICV-STZ animals [46, 47]. In order to examine whether INT-777 treatments overcome the STZ-induced cognitive impairment, we performed MWM and Y-maze tests.

**Figure 2:**
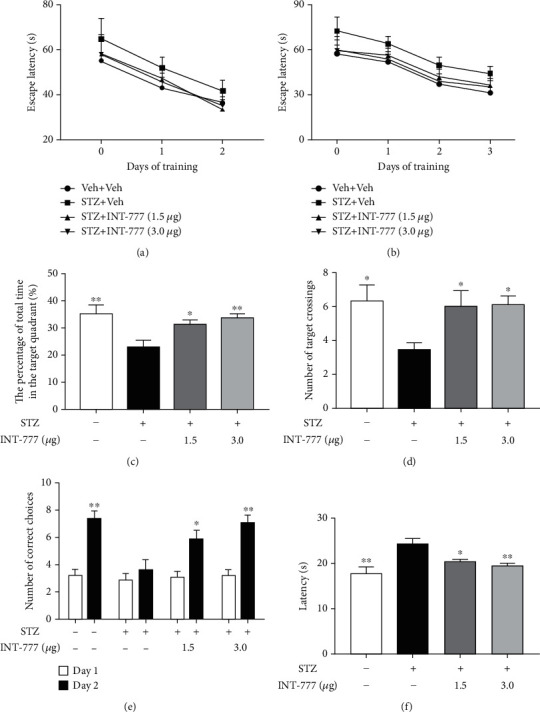
INT-777 ameliorates STZ-induced cognitive impairment in mice. (a) The mean escape latency to reach the visible platform in the MWM test. Day 0 indicates performance on the first trial, and subsequent points represent the average of all daily trials. (b) The mean escape latency to reach the hidden platform in the MWM test. (c) The percentage of time spent in the target quadrant. (d) The numbers of platform location crossings during the probe trial. (e) The number of correct choices on days 1-2 in the Y-maze test. (f) The latency to enter the shock-free compartment on day 2 in the Y-maze test. Values shown are expressed as the mean ± S.E.M.; *n* = 10 mice/group. ^∗^*P* < 0.05 and^∗∗^*P* < 0.01 vs. STZ+Veh group.

As shown in Figure 2(a), two-way ANOVA revealed that the escape latency did not differ among any of the groups during visible platform training in the MWM test, suggesting no influence of STZ or INT-777 on vision and basal movement of mice (4 trials/mouse/day for 2 days, effect of day, *F* [3, 340] = 12.56, *P* < 0.05; effect of group, *F* [3, 340] = 2.34, *P* > 0.05; and effect of group-by-day interaction, *F* [3, 340] = 5.69, *P* > 0.05; Figure 2(a)). During the hidden platform training (days 3-5), two-way ANOVA revealed that the escape latency did not significantly change among any of the groups (4 trials/d for 3 d, effect of day, *F* [3, 504] = 14.18, *P* < 0.05; effect of group, *F* [3, 504] = 3.26, *P* > 0.05; and effect of group-by-day interaction, *F* [3, 504] = 1.37, *P* > 0.05; Figure 2(b)). In the probe trial, the mice in the STZ+Veh group displayed a significant decrease in the percentage of time spent in the target quadrant (*P* < 0.01; Figure 2(c)) and the number of platform location crossings (*P* < 0.05; Figure 2(d)) compared with the control group, suggesting a spatial learning memory impairment in ICV-STZ mice. However, treatments of INT-777 significantly increased the percentage of time in the target quadrant (INT-777: 1.5 *μ*g/mouse, *P* < 0.05; 3.0 *μ*g/mouse, *P* < 0.01; Figure 2(c)) and the number of platform location crossings (INT-777: 1.5 and 3.0 *μ*g/mouse, *P* < 0.05; Figure 2(d)) compared with the STZ+Veh group.

Moreover, mouse spatial working memory was analyzed in the Y-maze test. One-way ANOVA revealed that mice in the STZ+Veh group showed significant decreases in the number of correct choices on day 2 (*F* [3, 36] = 5.56, *P* < 0.01; Figure 2(e)) and increases in latency to enter the shock-free compartment (*F* [3, 36] = 6.45, *P* < 0.01; Figure 2(f)) compared with the control group, suggesting spatial working memory impairment in ICV-STZ mice. However, treatments of INT-777 significantly increased the number of correct choices (INT-777: 1.5 *μ*g/mouse, *P* < 0.05; 3.0 *μ*g/mouse, *P* < 0.01; Figure 2(e)) and decreased the latency to enter the shock-free compartment (INT-777: 1.5 *μ*g/mouse, *P* < 0.05; 3.0 *μ*g/mouse, *P* < 0.01; Figure 2(f)) compared with the STZ+Veh group. Taken together, these results suggest that STZ-induced cognitive impairment, specifically a deficit in spatial memory, can be significantly ameliorated by TGR5 activation with INT-777.

### 3.3. INT-777 Alleviates STZ-Induced Decreases of TGR5 Expression in the Hippocampus and Frontal Cortex

**Figure 3:**
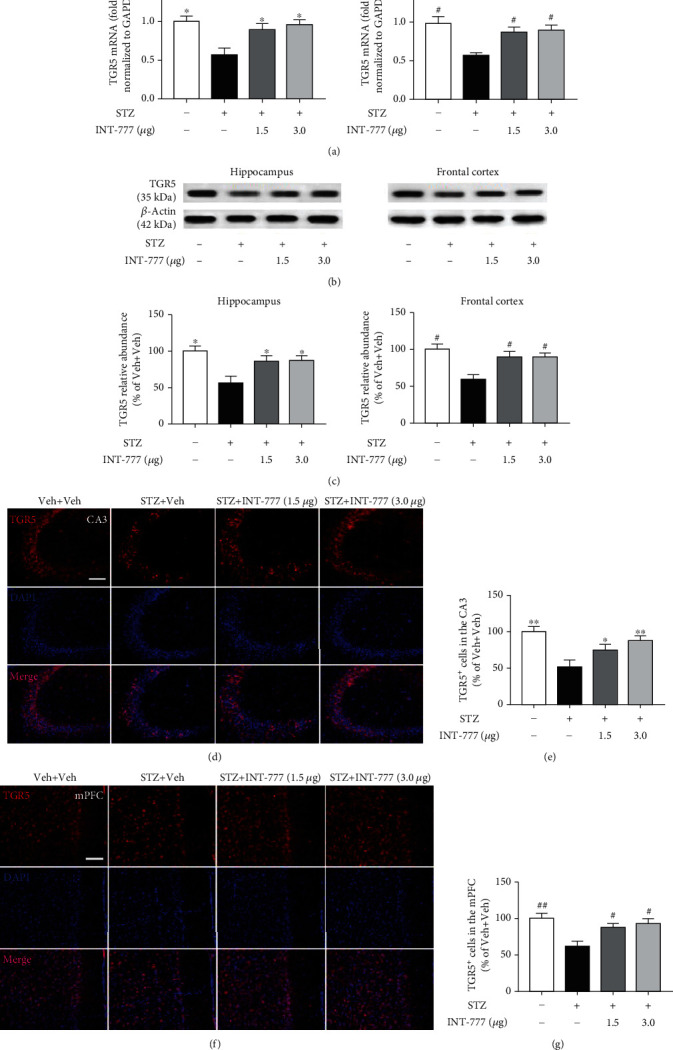
INT-777 alleviates STZ-induced decreases of TGR5 expression in the hippocampus and frontal cortex. (a) The level of TGR5 mRNA in the hippocampus and frontal cortex was detected by qRT-PCR. GAPDH was used as a loading control. (b) TGR5 expression of the hippocampus and frontal cortex was detected by western blot. *β*-Actin was used as a loading control. (c) Quantification of TGR5 level was expressed as the ratio (in percentage) of the Veh+Veh group. (d, e) TGR5-positive cells of the hippocampal CA3 were detected and quantified by immunofluorescence. (f, g) TGR5-positive cells of the mPFC were detected and quantified. Values shown are expressed as the mean ± S.E.M.; *n* = 3 mice/group in qRT-PCR and western blot; *n* = 4 mice/group, four sections per mouse in immunofluorescence. Scale bar: 100 *μ*m (d, f). ^∗^*P* < 0.05 and^∗∗^*P* < 0.01 vs. the hippocampus or CA3 of STZ+Veh group; ^#^*P* < 0.05 and ^##^*P* < 0.01 vs. the frontal cortex or mPFC of STZ+Veh group.

To further confirm the protective effects of INT-777 on STZ-induced cognitive deficits associated with TGR5, its mRNA level in the hippocampus and frontal cortex was detected by qRT-PCR, while its protein expression was detected by western blot. One-way ANOVA of the qRT-PCR assay showed that STZ injection induced significant decreases of TGR5 mRNA in the hippocampus and frontal cortex (*F* [3, 8] = 6.36, *P* < 0.05 for hippocampus; *F* [3, 8] = 7.05, *P* < 0.05 for frontal cortex; Figure 3(a)), which were reversed by INT-777 treatments (1.5 and 3.0 *μ*g/mouse: *P* < 0.05 for hippocampus and frontal cortex; Figure 3(a)). The data of western blot showed significant decreases of TGR5 in the hippocampus and frontal cortex in ICV-STZ mice (*F* [3, 8] = 6.18, *P* < 0.05 for hippocampus; *F* [3, 8] = 5.65, *P* < 0.05 for frontal cortex; Figures 3(b) and 3(c)), which were reversed by INT-777 treatments (1.5 and 3.0 *μ*g/mouse: *P* < 0.05 for hippocampus and frontal cortex; Figures 3(b) and 3(c)).

The CA3 region of the hippocampus is critical for the rapid encoding of memory [48]. In addition, the mPFC, as an important part of the frontal cortex, supports the retrieval of remote long-term memory and consolidation [49]. Our previous study has shown a large number of TGR5 in the hippocampal CA3 and mPFC [50]. To confirm the effects of STZ and INT-777 treatments on TGR5 expression in the CA3 and mPFC, an immunofluorescence assay was performed. One-way ANOVA showed significant decreases of TGR5 expression in the CA3 and mPFC in ICV-STZ mice (*F* [3, 12] = 12.19, *P* < 0.01 for CA3, Figures 3(d) and 3(e); *F* [3, 12] = 6.10, *P* < 0.01 for mPFC, Figures 3(f) and 3(g)), while INT-777 treatments alleviated this STZ-induced downregulation (INT-777: 1.5 *μ*g/mouse, *P* < 0.05; 3.0 *μ*g/mouse, *P* < 0.01 for CA3, Figures 3(d) and 3(e); 1.5 and 3.0 *μ*g/mouse, *P* < 0.05 for mPFC, Figures 3(f) and 3(g)). These results suggest that TGR5 might be involved in STZ-induced cognitive deficits.

### 3.4. TGR5 Activation by INT-777 Alleviates STZ-Induced Glucose Usage Deficits in the Hippocampus and Frontal Cortex

**Figure 4:**
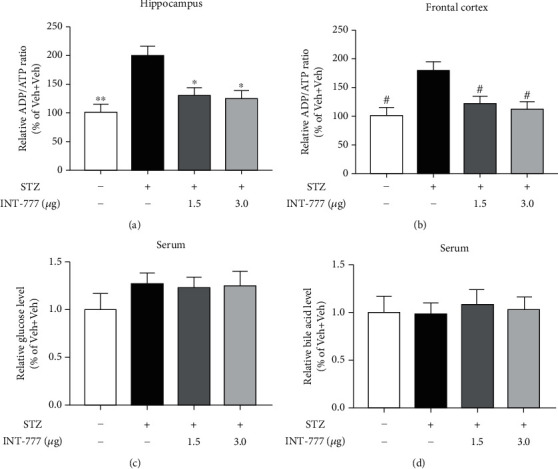
INT-777 alleviates STZ-induced glucose usage deficits in the hippocampus and frontal cortex. (a, b) Quantification of glucose usage was expressed as the ADP/ATP ratio (in percentage) of the Veh+Veh group in the hippocampus (a) and frontal cortex (b). (c, d) Quantification of glucose (c) and bile acid (d) in the serum. Values shown are expressed as the mean ± S.E.M.; *n* = 3 mice/group. ^∗^*P* < 0.05 and^∗∗^*P* < 0.01 vs. the hippocampus of the STZ+Veh group; ^#^*P* < 0.05 vs. the frontal cortex of the STZ+Veh group.

The functioning and survival of mammalian neurons require an active energy metabolism. The monosaccharide glucose which constitutes a key source of cellular energy is converted into pyruvate. Then, the metabolite ATP is generated mainly within the mitochondria following the process of oxidative phosphorylation [51]. ATP is consumed as a biological energy source by many intracellular reactions, while ADP is produced, and the ADP/ATP ratio has been used to detect the parameter of glucose usage and differentiate the different modes of cell death and viability [52, 53]. To explore whether INT-777 treatments alleviate the STZ-induced glucose usage deficits, the ADP/ATP ratio of neurons in the hippocampus and frontal cortex was detected. One-way ANOVA showed that STZ injection induced significant increases of the ADP/ATP ratio in the hippocampus and frontal cortex (*F* [3, 8] = 9.11, *P* < 0.01 for hippocampus, Figure 4(a); *F* [3, 8] = 7.43, *P* < 0.05 for frontal cortex, Figure 4(b)), which were reversed by INT-777 treatments (1.5 and 3.0 *μ*g/mouse: *P* < 0.05 for hippocampus and frontal cortex; Figures 4(a) and 4(b)). However, both STZ and INT-777 injections did not change the levels of glucose and bile acid in the serum (*F* [3, 8] = 0.951, *P* = 0.461 for serum glucose, Figure 4(c); *F* [3, 8] = 0.083, *P* = 0.967 for serum bile acid, Figure 4(d)). These results indicate that TGR5 activation by INT-777 alleviates glucose usage deficits in the hippocampus and frontal cortex rather than affecting the levels of serum glucose and bile acid.

### 3.5. TGR5 Activation by INT-777 Alleviates STZ-Induced Apoptosis and Tau Hyperphosphorylation in the Hippocampus and Frontal Cortex

**Figure 5:**
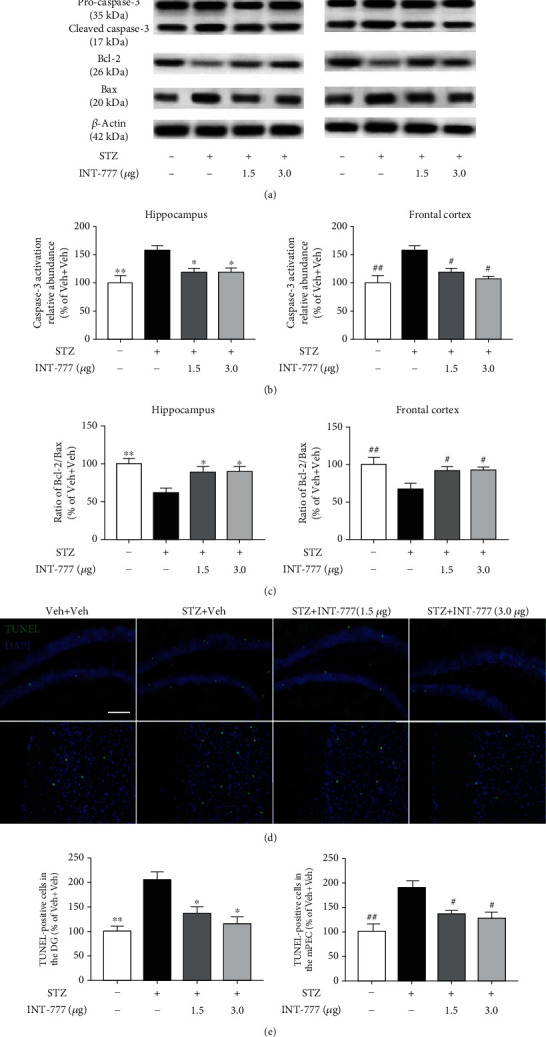
INT-777 alleviates STZ-induced apoptosis in the hippocampus and frontal cortex. (a) Representative immunoblots of pro-caspase-3, cleaved caspase-3, Bcl-2, and Bax in the hippocampus and frontal cortex. (b) Quantification of caspase-3 activation with the ratio of cleaved caspase-3/pro-caspase-3. (c) Quantification of tau hyperphosphorylation with the ratio of Bcl-2/Bax. *β*-Actin was used as a loading control. (d) Representative image of TUNEL-positive cells in the hippocampal DG and mPFC. Scale bar: 100 *μ*m. (e) Quantification of TUNEL-positive cells was expressed as the ratio (in percentage) of the Veh+Veh group. Values shown are expressed as the mean ± S.E.M.; *n* = 3 mice/group for western blot and TUNEL staining. ^∗^*P* < 0.05 and^∗∗^*P* < 0.01 vs. the hippocampus or DG of STZ+Veh group; ^#^*P* < 0.05 and ^##^*P* < 0.01 vs. the frontal cortex or mPFC of STZ+Veh group.

The excessive apoptosis of neurons in the hippocampus and frontal cortex leads to cognitive impairment [54]. The antiapoptotic effects of INT-777 were also investigated by detecting apoptotic-related proteins. The cleaved caspase-3 is an active caspase-3 form. Results of western blot showed that the expression of active caspase-3 was induced by STZ but decreased by INT-777 treatments both in the hippocampus and frontal cortex, while the pro-caspase-3 level was not changed in each group (Figure 5(a)). In addition, the expression of antiapoptotic protein Bcl-2 was less in the STZ+Veh group than that of the control group but improved after INT-777 treatments, while the proapoptotic protein Bax showed the opposite way (Figure 5(a)). One-way ANOVA showed that the ratio of cleaved caspase-3/pro-caspase-3 in the hippocampus and frontal cortex was significantly increased while the ratio of Bcl-2/Bax was declined with STZ injection (cleaved caspase-3/pro-caspase-3: *F* [3, 8] = 6.36, *P* < 0.01 for the hippocampus, *F* [3, 8] = 6.11, *P* < 0.01 for the frontal cortex, Figure 5(b); Bcl-2/Bax: *F* [3, 8] = 7.50, *P* < 0.01 for the hippocampus, *F* [3, 8] = 6.54, *P* < 0.01 for the frontal cortex, Figure 5(c)) but reversed after INT-777 treatments (cleaved caspase-3/pro-caspase-3 and Bcl-2/Bax, *P* < 0.05 for the hippocampus and frontal cortex, Figures 5(b) and 5(c)). We further performed TUNEL staining in the dentate gyrus (DG) and mPFC. The results showed that ICV injection of STZ induced significant increases of apoptotic cells (*F* [3, 8] = 9.22, *P* < 0.01 for hippocampus, *F* [3, 8] = 8.95, *P* < 0.01 for frontal cortex; Figures 5(d) and 5(e)), which were reversed by INT-777 treatments (1.5 and 3.0 *μ*g/mouse: *P* < 0.05 for hippocampus and frontal cortex; Figures 5(d) and 5(e)). Moreover, INT-777 alleviated STZ-induced decreases of the neuronal population (Supplementary Figure 1A and B). Together, these results reveal that TGR5 activation by INT-777 alleviates STZ-induced apoptosis in the hippocampus and frontal cortex.

**Figure 6:**
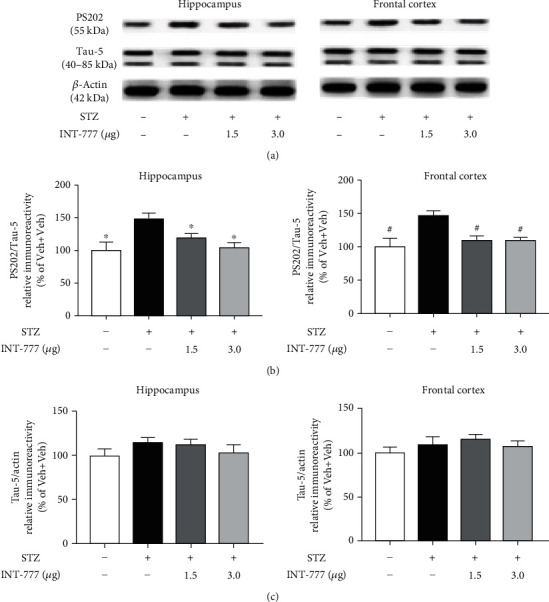
INT-777 alleviates STZ-induced tau hyperphosphorylation in the hippocampus and frontal cortex. (a) Representative immunoblots of tau hyperphosphorylation at Ser202 (PS202) and total tau (Tau5) in the hippocampus and frontal cortex. (b) Quantification of the ratio of PS202/Tau5 in western blot. (c) Quantification of Tau5 expression in western blot. *β*-Actin was used as a loading control. Values shown are expressed as the mean ± S.E.M.; *n* = 3 mice/group. ^∗^*P* < 0.05 vs. the hippocampus of the STZ+Veh group; ^#^*P* < 0.05 vs. the frontal cortex of the STZ+Veh group.

Abnormal hyperphosphorylation of tau is one of the most important pathophysiological features in AD and is associated with neurodegeneration and apoptosis [55]. To evaluate whether INT-777 treatments ameliorate the accumulation of phosphorylated tau in ICV-STZ mice, western blot was performed. As expected, a marked increase in tau hyperphosphorylation at the site of Ser202 was observed in the STZ+Veh group compared with the control mice (*F* [3, 8] = 4.17, *P* < 0.05 for the hippocampus, *F* [3, 8] = 4.52, *P* < 0.05 for the frontal cortex; Figures 6(a) and 6(b)). Excitingly, INT-777 treatments attenuated the effects of STZ (*P* < 0.05 for the hippocampus and frontal cortex, Figures 6(a) and 6(b)). However, there is no significant change in the total tau (Tau5) level in each group of mice (*F* [3, 8] = 0.93, *P* > 0.05 for the hippocampus, *F* [3, 8] = 0.26, *P* > 0.05 for the frontal cortex; Figures 6(a) and 6(c)). We further showed that the neuroprotective effects against tau hyperphosphorylation were involved in the GSK3*β* signaling (Supplementary Figure 2A and B). These results indicate that TGR5 activation by INT-777 alleviates STZ-induced tau hyperphosphorylation in the hippocampus and frontal cortex.

### 3.6. TGR5 Activation by INT-777 Alleviates STZ-Induced Decreases of Neurogenesis in the Hippocampal DG

**Figure 7:**
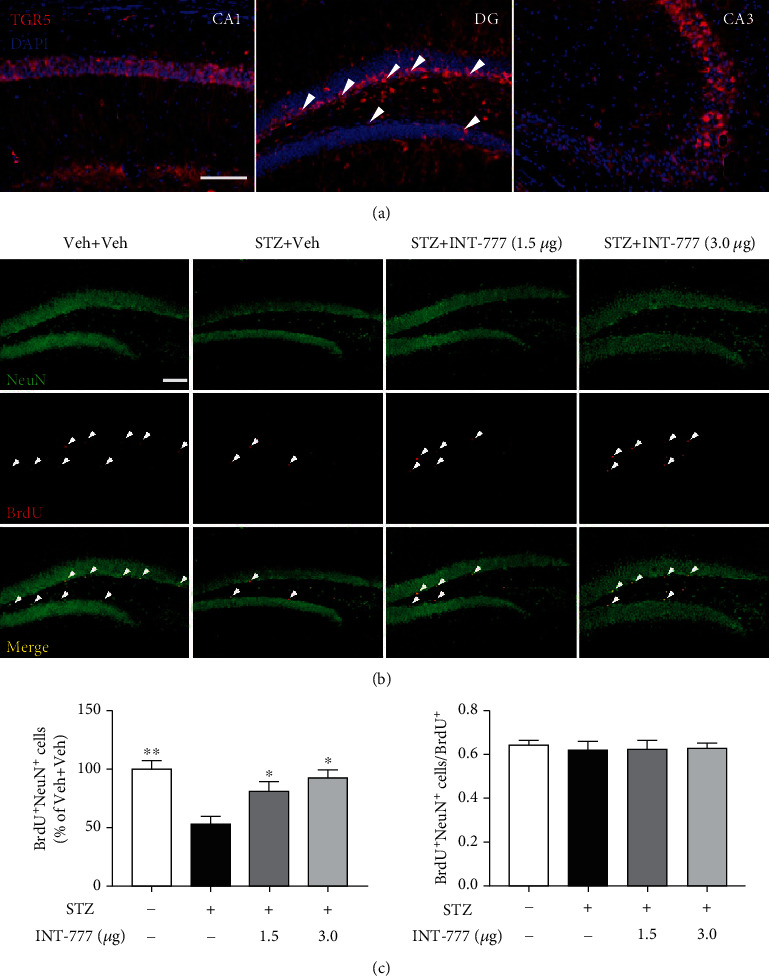
INT-777 alleviates STZ-induced decreases of neurogenesis in the DG of hippocampus. (a) TGR5-positive cells of the hippocampal CA1, DG, and CA3 were detected by immunofluorescence. TGR5^+^ cells in the SGZ of hippocampal DG were marked with white arrows. (b) Neurogenesis in the hippocampal DG was detected by immunofluorescence of BrdU and NeuN. TGR5^+^BrdU^+^ cells in the SGZ were marked with white arrows. (c) Quantification of the colabeled BrdU^+^ and NeuN^+^ cells. Values shown are expressed as the mean ± S.E.M.; *n* = 3 mice/group, four sections per mouse. ^∗^*P* < 0.05 and^∗∗^*P* < 0.01 vs. the hippocampal DG of STZ+Veh group. Scale bar: 100 *μ*m (a, b).

Accumulation of phosphorylated tau impairs adult hippocampal neurogenesis [56]. The impairment of hippocampal neurogenesis at the early stages of AD is believed to support early cognitive decline [57]. Here, we showed that TGR5 was mainly expressed in the pyramidal neurons of hippocampal CA1 and CA3 and in the granule cells of the DG subgranular zone (SGZ) (Figure 7(a)). Neurogenesis in the brain occurs throughout life in the ventricular-subventricular zone of the lateral ventricle and the SGZ of hippocampal DG [58]. BrdU staining was carried out 16 d after the last BrdU injection in mice, by which time newborn cells developed differentiated phenotypes. To examine the phenotype of BrdU-positive cells in the SGZ of DG, double labeling for BrdU and NeuN (a neuronal marker) was performed. The date showed that the number of BrdU^+^NeuN^+^ cells in the SGZ of the ICV-STZ group significantly decreased relative to the control mice (*F* [3, 8] = 8.36, *P* < 0.01; Figures 7(b) and 7(c)). However, the number of BrdU^+^NeuN^+^ cells in the SGZ in mice treated with INT-777 was significantly increased compared with ICV-STZ mice (*P* < 0.05 for INT-777; Figures 7(b) and 7(c)). These data suggest that TGR5 activation by INT-777 enhances hippocampal neurogenesis in ICV-STZ mice.

### 3.7. TGR5 Activation by INT-777 Alleviates STZ-Induced Downregulation of CREB-BDNF Signaling in the Hippocampus and Frontal Cortex

**Figure 8:**
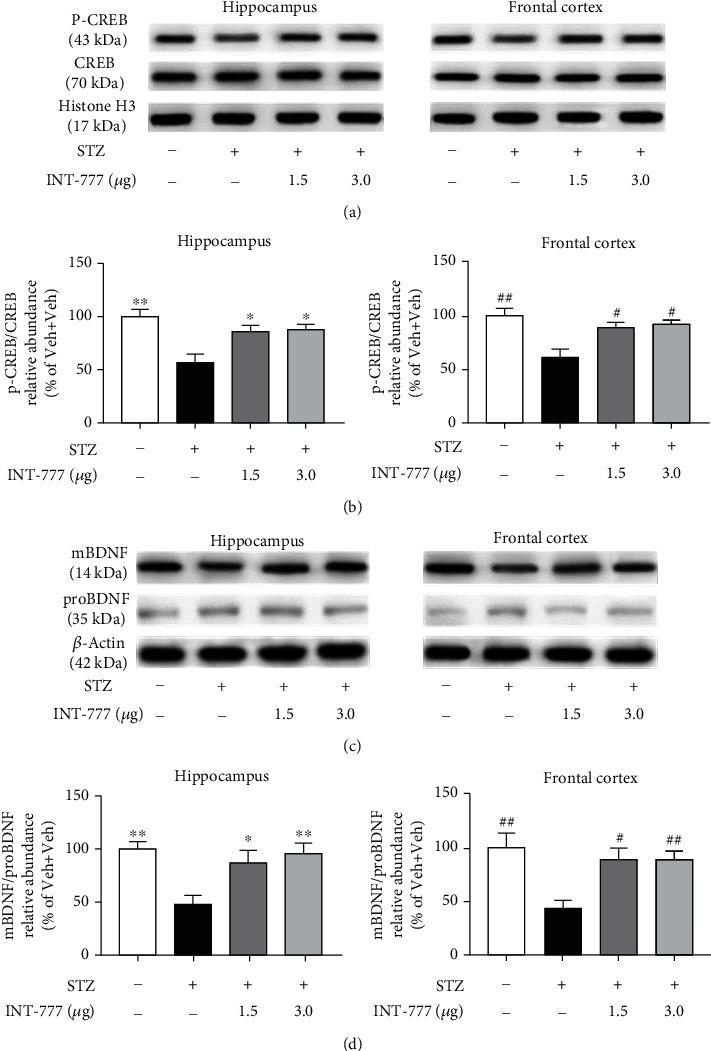
INT-777 alleviates STZ-induced downregulation of CREB-BDNF signaling in the hippocampus and frontal cortex. (a, b) Representative immunoblots of p-CREB and CREB (a) and quantification of the ratio of p-CREB/CREB (b). (c, d) Representative immunoblots of mBDNF and proBDNF (c) and quantification of the ratio of mBDNF/pro-BDNF (d). Histone H3 or *β*-actin was used as a loading control, respectively. Values shown are expressed as the mean ± S.E.M.; *n* = 3 mice/group. ^∗^*P* < 0.05 and^∗∗^*P* < 0.01 vs. the hippocampus of the STZ+Veh group; ^#^*P* < 0.05 and ^##^*P* < 0.01 vs. the frontal cortex of STZ+Veh group.

The CREB-BDNF pathway is critical for hippocampal neurogenesis and apoptosis which are closely involved in the pathogenesis of AD [59]. To evaluate whether INT-777 plays a role in the CREB-BDNF signaling, the expression of CREB and BDNF in the hippocampus and frontal cortex was examined. One-way ANOVA showed significant decreases of the ratio of p-CREB/CREB and mBDNF/pro-BDNF in the STZ+Veh group compared with the control group (p-CREB/CREB: *F* [3, 8] = 8.14, *P* < 0.01 for the hippocampus, *F* [3, 8] = 7.35, *P* < 0.01 for the frontal cortex, Figures 8(a) and 8(b); mBDNF/pro-BDNF: *F* [3, 8] = 6.72, *P* < 0.01 for the hippocampus, *F* [3, 8] = 5.71, *P* < 0.01 for the frontal cortex; Figures 8(c) and 8(d)). However, these changes were reversed by the treatments of INT-777 (p-CREB/CREB: *P* < 0.01 (INT-777 1.5 and 3.0 *μ*g) for the hippocampus and frontal cortex, Figures 8(a) and 8(b); mBDNF/pro-BDNF: *P* < 0.05 (INT-777 1.5 *μ*g) and *P* < 0.01 (INT-777 3.0 *μ*g) for the hippocampus and frontal cortex, Figures 8(c) and 8(d)). These results demonstrate that TGR5 activation by INT-777 alleviates STZ-induced downregulation of CREB-BDNF signaling in the hippocampus and frontal cortex.

### 3.8. TGR5 Activation by INT-777 Ameliorates STZ-Induced Decreases of Action Potential Firing of Excitatory Pyramidal Neurons in the Hippocampal CA3 and mPFC

**Figure 9:**
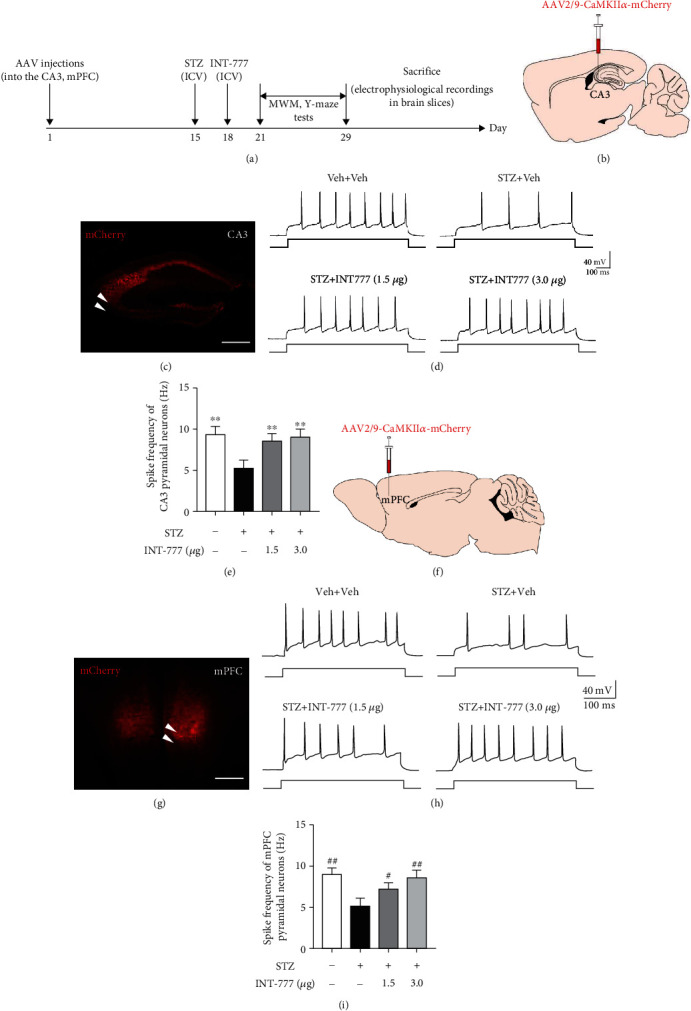
INT-777 ameliorates STZ-induced decreases of action potential firing of excitatory pyramidal neurons in the hippocampal CA3 and mPFC. (a) Schematic illustrating the timeline for virus injection, drug administration, behavioral tests, and electrophysiological recordings. (b, c) Schematic diagram depicting virus injection (b) and representative image of AAV-labeled excitatory pyramidal neurons in the hippocampal CA3 (c). (d, e) Representative traces of action potential firing (d) and quantification of the frequency of firing (e) of CA3 neurons with depolarizing current step (+70 pA). (f, g) Schematic diagram depicting virus injection (f) and representative image of AAV-labeled excitatory pyramidal neurons in the mPFC (g). (h, i) Representative traces of action potential firing (h) and quantification of the frequency of firing (i) of mPFC neurons with depolarizing current step (+70 pA). Scale bar: 400 *μ*m (c, g). Values shown are expressed as mean ± S.E.M.; *n* = 4 mice/group, 3 cells analyzed per mouse. ^∗∗^*P* < 0.01 vs. the CA3 of STZ+Veh group; ^#^*P* < 0.05 and ^##^*P* < 0.01 vs. the mPFC of STZ+Veh group.

Firing of excitatory pyramidal neurons in the hippocampus and mPFC is often associated with memories [60]. To confirm whether STZ results in dysfunction of neuronal firing and TGR5 activation by INT-777 has a neuroprotective effect, another separate set of groups of C57BL/6 mice were specially used for electrophysiological recordings in brain slices (Figure 9(a)). Excitatory pyramidal neurons in the hippocampal CA3 and mPFC were marked with AAV2/9-CaMKII*α*-mCherry vector before STZ and INT-777 injections (Figures 9(b), 9(c), 9(f), and 9(g)), and the action potential firing was recorded by whole-cell patch-clamp after behavioral tests. The results of MWM and Y-maze tests showed a success of the ICV-STZ model and treatment of INT-777 (Supplementary Figure 3A and B). One-way ANOVA showed that STZ injection induced significant decreases in the frequency of action potential firing of excitatory pyramidal neurons (*F* [3, 12] = 14.94, *P* < 0.01 for CA3, Figures 9(d) and 9(e); *F* [3, 12] = 15.55, *P* < 0.01 for mPFC; Figures 9(h) and 9(i)), which were significantly ameliorated by INT-777 treatments (1.5 and 3.0 *μ*g/mouse: *P* < 0.01 for CA3, Figures 9(d) and 9(e); 1.5 *μ*g/mouse, *P* < 0.05; 3.0 *μ*g/mouse, *P* < 0.01 for mPFC, Figures 9(h) and 9(i)), suggesting TGR5 activation by INT-777 could improve action potential firing of excitatory pyramidal neurons in the hippocampal CA3 and mPFC.

### 3.9. TGR5 Activation by INT-777 Stimulates Excitatory Pyramidal Neuron Firing in the Hippocampal CA3 and mPFC Not due to Postsynaptic Response

**Figure 10:**
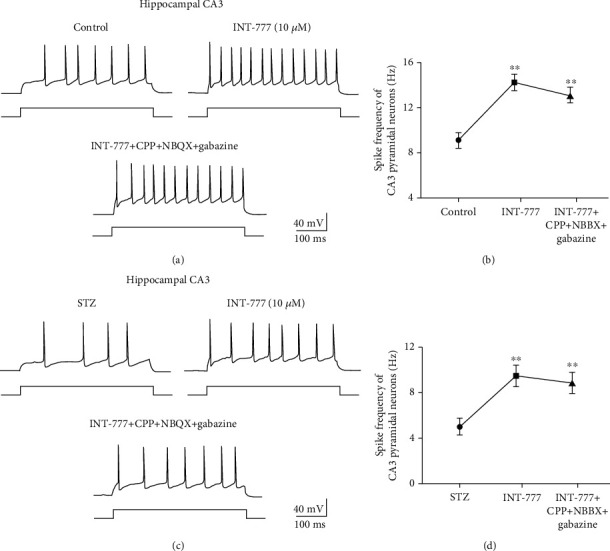
INT-777 activates excitatory pyramidal neurons in the hippocampal CA3 not due to postsynaptic response. (a, b) Representative traces of action potential firing (a) and quantification of the frequency of firing (b) of hippocampal CA3 pyramidal neurons with depolarizing current step (+70 pA) in control mice. (c, d) Representative traces of action potential firing (c) and quantification of the frequency of firing (d) of hippocampal CA3 pyramidal neurons with depolarizing current step (+70 pA) in ICV-STZ mice. Values shown are expressed as the mean ± S.E.M.; *n* = 4 mice/group, 3 cells analyzed per mouse. ^∗∗^*P* < 0.01 vs. control or ICV-STZ group.

**Figure 11:**
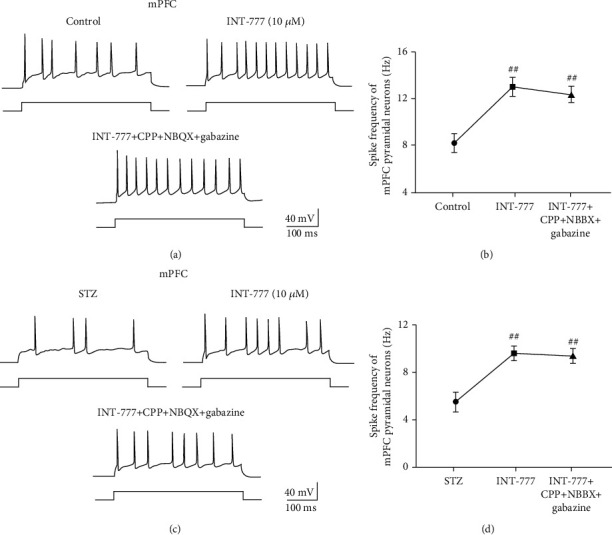
INT-777 activates excitatory pyramidal neurons in the mPFC not due to postsynaptic response. (a, b) Representative traces of action potential firing (a) and quantification of the frequency of firing (b) of mPFC pyramidal neurons with depolarizing current step (+70 pA) in control mice. (c, d) Representative traces of action potential firing (c) and quantification of the frequency of firing (d) of mPFC pyramidal neurons with depolarizing current step (+70 pA) in ICV-STZ mice. Values shown are expressed as the mean ± S.E.M.; *n* = 4 mice/group, 3 cells analyzed per mouse. ^##^*P* < 0.01 vs. control or ICV-STZ group.

To explore the underlying mechanism of TGR5 activation by INT-777 improving action potential firing of excitatory pyramidal neurons in the hippocampal CA3 and mPFC, the ionotropic glutamate receptor antagonists (CPP and NBQX) and the GABA_A_ receptor antagonist (gabazine) were used to block presynaptic roles of other glutamatergic and GABAergic neurons in whole-cell patch-clamp recording. One-way ANOVA showed that bath application of TGR5 agonist INT-777 (10 *μ*M) significantly increased frequency of action potential firing of hippocampal CA3 excitatory pyramidal neurons in control and ICV-STZ mice (*F* [2, 8] = 10.53, *P* < 0.01 for control mice, Figures 10(a) and 10(b); *F* [2, 8] = 8.28, *P* < 0.01 for ICV-STZ mice, Figures 10(c) and 10(d)). Moreover, the neuronal activation role of INT-777 was not blocked following bath application of a mixture of CPP, NBQX, and gabazine (*P* < 0.01 for control and ICV-STZ mice, Figures 10(a)–10(d)). With the same approach, one-way ANOVA showed that bath application of INT-777 (10 *μ*M) significantly increased the frequency of action potential firing of mPFC excitatory pyramidal neurons in control and ICV-STZ mice (*F* [2, 8] = 11.49, *P* < 0.01 for control mice, Figures 11(a) and 11(b); *F* [2, 8] = 8.28, *P* < 0.01 for ICV-STZ mice, Figures 11(c) and 11(d)), and glutamate and GABA_A_ receptor antagonists did not block this neuronal activation effect (*P* < 0.01 for control and ICV-STZ mice, Figures 11(a)–11(d)). These results reveal that INT-777 activates excitatory pyramidal neurons in the hippocampal CA3 and mPFC not due to postsynaptic response.

## 4. Discussion

AD can be divided into SAD and FAD. SAD, caused by multiple etiologic factors, including environmental, genetic, and metabolic factors, is the majority of AD cases in the clinic [4]. STZ cytotoxicity is mainly due to DNA alkylation which results in cellular necrosis. Single or double ICV-STZ injection(s) chronically decrease cerebral glucose utilization and produce multiple other effects that resemble molecular, pathological, and behavioral features of SAD [47]. Potential drugs for SAD can be evaluated preclinically in the nontransgenic model of ICV-STZ mouse [11]. Consistent with previous findings, our study showed significant decreases in the time spent on the goal quadrant and the number of goal crossings in the MWM test in ICV-STZ mice while an increase in the latency to enter the shock-free compartment in the Y-maze test, indicating that STZ injection leads to cognitive impairment in mice. However, INT-777, a specific TGR5 agonist, which was injected into the lateral ventricle of the brain, ameliorated STZ-induced cognitive deficits, suggesting that TGR5 might be a novel and potential target for AD.

Glucose hypometabolism and energy deficit are hallmarks of AD, and previous studies have showed that reduced glucose utilization in brain regions affects patients with AD [14]. The monosaccharide glucose constitutes a key source of cellular energy. Following its import across the plasma membrane, glucose is converted into pyruvate by the glycolysis pathway. Pyruvate oxidation supplies substrates for the ATP-generating mitochondrial oxidative phosphorylation system [51]. In the process of phosphoryl transfer from ATP, the ADP is produced, and as a result, the ADP-to-ATP ratio is an important physiological control parameter [61–63]. Although STZ is an antibiotic that produces pancreatic islet *β*-cell destruction and is widely used experimentally to produce a model of diabetes mellitus [6, 7], we showed that intracerebroventricular injection of STZ could not affect the level of blood glucose in mice. But there was a glucose usage deficit in ICV-STZ mice by detecting the ADP/ATP ratio of neurons in the hippocampus and frontal cortex. The previous study has demonstrated that the bile acid membrane receptor TGR5 can regulate energy homeostasis and glucose metabolism in cells in vitro [64]. In this study, TGR5 activation by INT-777 significantly improved glucose usage of neurons in the hippocampus and frontal cortex, suggesting that TGR5 plays important roles in maintaining energy homeostasis in the brain. In addition, bile acids are signal molecules which can reach the brain through the blood-brain barrier and mediate various cellular responses in both physiological and pathological processes [65]. In the ICV-STZ model, intraperitoneal injection of bile acid for a long period not only improves STZ-induced cognitive deficits and glucose metabolism but also increases glucose-stimulated insulin secretion and *β*-cell number [66]. Here, we further showed that activation of TGR5 in the brain has neuroprotective effects on glucose metabolism, which was not due to the change of the bile acid level in the blood.

Cellular longevity is associated with apoptosis and neurogenesis, which are associated with energy metabolism. Decreased levels of ATP and increased levels of ADP are recognized in apoptotic cells [67]. Apoptosis is a programmed form of cell death controlled by genes, and activation of such genes may be caused by environmental stimuli including DNA damage, oxidative stress, exposure to hormones, drugs, toxins, virus, and withdrawal of trophic supports [68]. The biochemical events of apoptosis are triggered by a family of cysteine proteases called caspases. One main player in apoptotic cell death is caspase-3 as shown in various paradigms of neuronal cell death and neurodegeneration [69]. In addition, proapoptotic protein Bax and antiapoptotic protein Bcl-2 are involved in regulating apoptosis [70, 71]. AD is characterized by severe neuronal apoptosis, and drugs reduce neuronal apoptosis to improve memory in AD model animals [72]. Previous studies have shown that STZ-induced memory impairment is associated with decreased Bcl-2 expression and Bcl-2/Bax ratio [73]. In our study, STZ enhanced caspase-3 activation and decreased Bcl-2 in the hippocampus and frontal cortex, while TGR5 activation by INT-777 exhibited positive effects on apoptosis by decreasing caspase-3 activity and increasing the ratio of Bcl-2/Bax. Moreover, STZ-induced apoptotic responses and INT-777 playing antiapoptotic roles were confirmed by TUNEL staining in hippocampal DG and mPFC. Tau is a microtubule-binding protein, and hyperphosphorylation of tau contributes to neurodegeneration and influences apoptosis [74]. It is recognized that tau hyperphosphorylation is one of the main features of neurodegeneration in AD [75]. Here, we evaluated the effects of TGR5 activation by INT-777 on tau hyperphosphorylation in ICV-STZ mice. We found that TGR5 activation attenuated the hyperphosphorylation of tau at the site of Ser202, providing the first evidence for a potential role of TGR5 in STZ-induced tau hyperphosphorylation. We also showed that the mechanism of inhibition of tau hyperphosphorylation by INT-777 was involved in the GSK3*β* pathway.

Structural plasticity and network maintenance of the hippocampus rely on neurogenesis, and increased levels of ATP and decreased levels of ADP have been recognized in proliferating cells. Dysfunctional neurogenesis resulting from early subtle disease manifestations may exacerbate neuronal vulnerability to AD and contribute to memory impairment [18]. Recent evidence has shown that interneuron accumulation of phosphorylated tau impairs adult hippocampal neurogenesis by suppressing GABAergic transmission [56], and tau accumulation of the hippocampal DG alters mitochondrial dynamics and function which leads to a reduction of adult neurogenesis [76]. Hippocampal neurogenesis in the brain occurs throughout life in the subgranular zone of the DG [58], and there was a significant decrease in hippocampal neurogenesis in ICV-STZ mice [77]. In the current study, we showed that activation of TGR5 enhanced neurogenesis in the hippocampal DG. Taken together, our results suggest that TGR5 activation ameliorates STZ-induced cognitive impairment partly due to an improvement of cellular longevity that involved modulating apoptosis and neurogenesis.

The cAMP response element binding protein (CREB) is at a central converging point of pathways and mechanisms activated during the processes of synaptic strengthening and memory formation [78]. CREB signaling influences cognitive processes directly by affecting memory and indirectly by affecting adult hippocampal neurogenic capacity [79]. BDNF influences the neuronal synaptic plasticity and facilitates hippocampal long-term potentiation (LTP) [80]. The activation of the CREB-BDNF pathway protects brain neurons against oxidative stress and apoptosis and promotes neurogenesis [81]. In AD, there is a significant decrease in the levels of CREB-regulated mBDNF, and disruption of the pathway is correlated with cognitive decline [82, 83]. Importantly, agents with potential therapeutic worth for AD often have enhancing effects on the CREB-BDNF pathway [84]. Our study showed a decrease of CREB phosphorylation and BDNF maturation of the hippocampus and frontal cortex in ICV-STZ mice while TGR5 activation by INT-777 reversed these changes, suggesting that the CREB-BDNF pathway might play key roles in improving hippocampal neurogenesis under activation of TGR5 in the SAD model of ICV-STZ. Additionally, some studies have shown that ICV-STZ mice can be observed with hyperinflammatory signal signs [85], and our previous studies have confirmed that activation of TGR5 by INT-777 has anti-inflammatory effects in A*β*_1–42_-induced or lipopolysaccharide-induced AD models [35, 86]. In this study, we also revealed that activation of TGR5 by INT-777 decreased NF-*κ*B signaling and production of proinflammatory cytokines TNF-*α* and IL-1*β* while suppressing microglia activation in ICV-STZ mice (Supplementary Figures 4-6).

Neuronal firing homeostasis is a master regulator of the integrative homeostatic network that maintains the stability of neural circuits and safeguards from neurodegeneration [23]. Extracellular ATP and energy are powerful triggers of neuronal firing [87]. Although numerous factors initiating AD have been extensively studied, the common principles underlying the transition from neuronal firing instability to homeostasis network collapse remain unknown. We showed that TGR5 was mainly expressed in excitatory pyramidal neurons of the CA1 and CA3 regions or granule cells of the DG subgranular zone. Because hippocampal CA3 pyramidal neurons which receive projections from the DG and put projections to the CA1 play key roles in memory [88], we evaluated whether TGR5 activation by INT-777 ameliorated the changes of firing patterns of CA3 pyramidal neurons in ICV-STZ mice. In this study, STZ injection resulted in a decrease in the frequency of action potential firing, suggesting dysfunction of neuronal firing of CA3 pyramidal neurons. With a bath application of INT-777, the decreased neuronal firing was reversed. In addition, the frontal cortex especially for mPFC is one of the key brain regions modulating memory coding and retrieval [89]. Recently, neuronal subpopulations exclusively containing excitatory neurons encode short-term memory in the prefrontal cortex [90], and pyramidal neuron activity in mouse mPFC during the delay period contributes to learning of a working memory task [91]. Here, we also explored the firing patterns of mPFC pyramidal neurons in ICV-STZ mice and showed that TGR5 activation by INT-777 ameliorated the dysfunction of mPFC neuronal firing. In particular, mPFC likely relies on the hippocampus to support memory encoding and consolidation [49]. Hippocampus also receives neuronal projections from other regions of the brain. For example, GABAergic medial septal neurons with low-rhythmic firing innervate hippocampal CA3 [92], while selective suppression of PV neurons in the medial septum projecting to CA3 impairs spatial working memory [93]. However, hippocampal CA3 receives glutamatergic projections mainly from the DG. Moreover, thalamic projections sustain activity of the prefrontal cortex during working memory maintenance [94], and the basolateral amygdala and mPFC forming strong reciprocal synaptic connections support acquisition and extinction of emotional memories [95]. To explore whether the neuroprotective effects of TGR5 are associated with neural circuits of CA3 and mPFC, we selectively blocked the glutamatergic and GABAergic projections using antagonists CPP, NBQX, and gabazine and showed that activation of TGR5 stimulated excitatory pyramidal neuron firing in the hippocampal CA3 and mPFC not due to postsynaptic response.

## 5. Conclusion

Our results suggest that activation of TGR5 by INT-777 ameliorates STZ-induced cognitive impairment by modulating apoptosis, neurogenesis, and neuronal firing, which might provide new insight into the mechanism of AD and highlight TGR5 as a novel and promising target for the prevention or treatment of AD.

## Data Availability

The datasets generated for this study are available upon request to the corresponding author.

## Supplementary Materials

Supplementary Materials INT-777 alleviates STZ-induced decreases of neuronal population (Supplementary Figure 1), phosphorylation of GSK3*β* (Supplementary Figure 2), cognitive impairment of C57BL/6 mice (Supplementary Figure 3), increases of NF-*κ*B signaling (Supplementary Figure 4) and proinflammatory cytokines (Supplementary Figure 5), and activation of microglia (Supplementary Figure 6).
