# Supplementary material for: Activation of TGR5 Ameliorates Streptozotocin-Induced Cognitive Impairment by Modulating Apoptosis, Neurogenesis, and Neuronal Firing
Source: Oxid Med Cell Longev. 2022 Apr 15;2022:3716609. doi: 10.1155/2022/3716609 (PMC9033389; doi:10.1155/2022/3716609)
Supplement: Supplementary Materials — INT-777 alleviates STZ-induced decreases of neuronal population (Supplementary Figure 1), phosphorylation of GSK3β (Supplementary Figure 2), cognitive impairment of C57BL/6 mice (Supplementary Figure 3), increases of NF-κB signaling (Supplementary Figure 4) and proinflammatory cytokines (Supplementary Figure 5), and activation of microglia (Supplementary Figure 6). [file 3716609.f1.docx]

**Supplementary Materials**

**B**


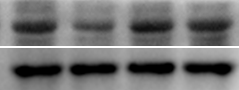


**NeuN**

**(46 kDa)**

**β-actin**

**(42 kDa)**

**A**


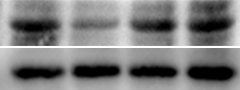


**Hippocampus**

**Frontal cortex**


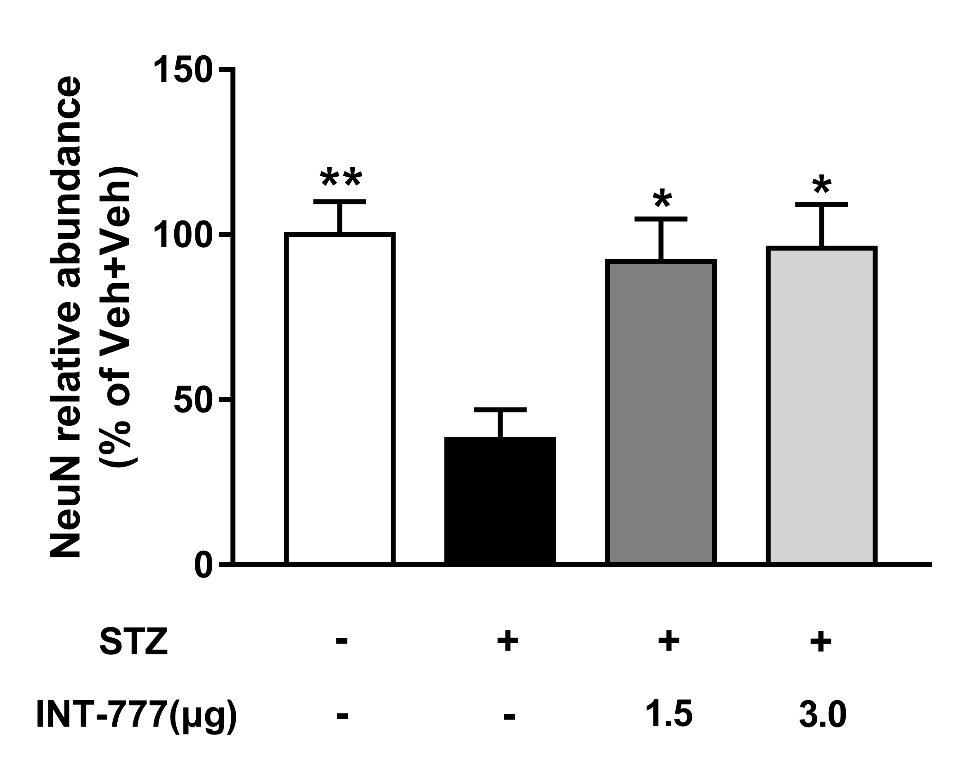


**Hippocampus**


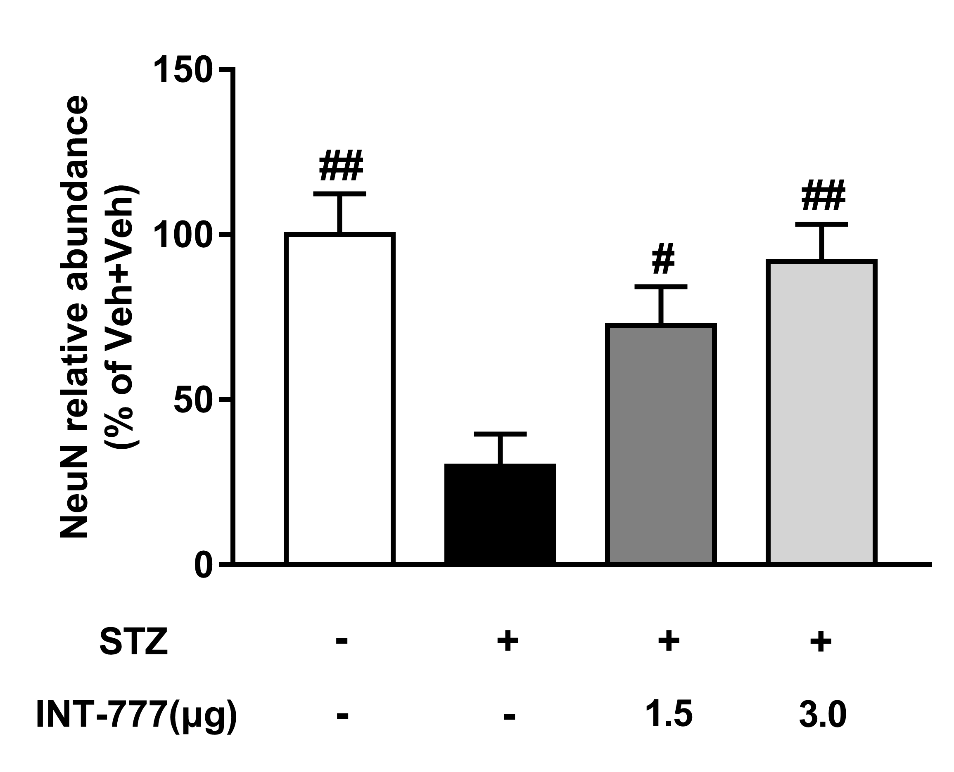


**Frontal cortex**

**Fig.** **S1** INT-777 alleviates STZ-induced decreases of neuronal population in the hippocampus and frontal cortex. (**A**) NeuN levels of the hippocampus and frontal cortex were detected by western blot. β-actin was used as a loading control. (**B**) Quantification of NeuN expression was expressed as the ratio (in percentage) of Veh + Veh group. Values shown are expressed as mean ± S.E.M; n = 3 mice/group. ^*^*P* < 0.05, ^**^*P* < 0.01 vs. the hippocampus of STZ + Veh group; ^#^*P* < 0.05, ^##^*P* < 0.01 vs. the frontal cortex of STZ + Veh group.


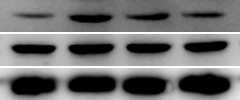

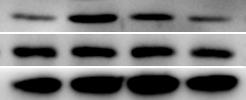


**P-GSK3β**

**(46 kDa)**

**β-actin**

**(42 kDa)**

**GSK3β**

**(46 kDa)**

**Hippocampus**

**Frontal cortex**

**A**

**B**


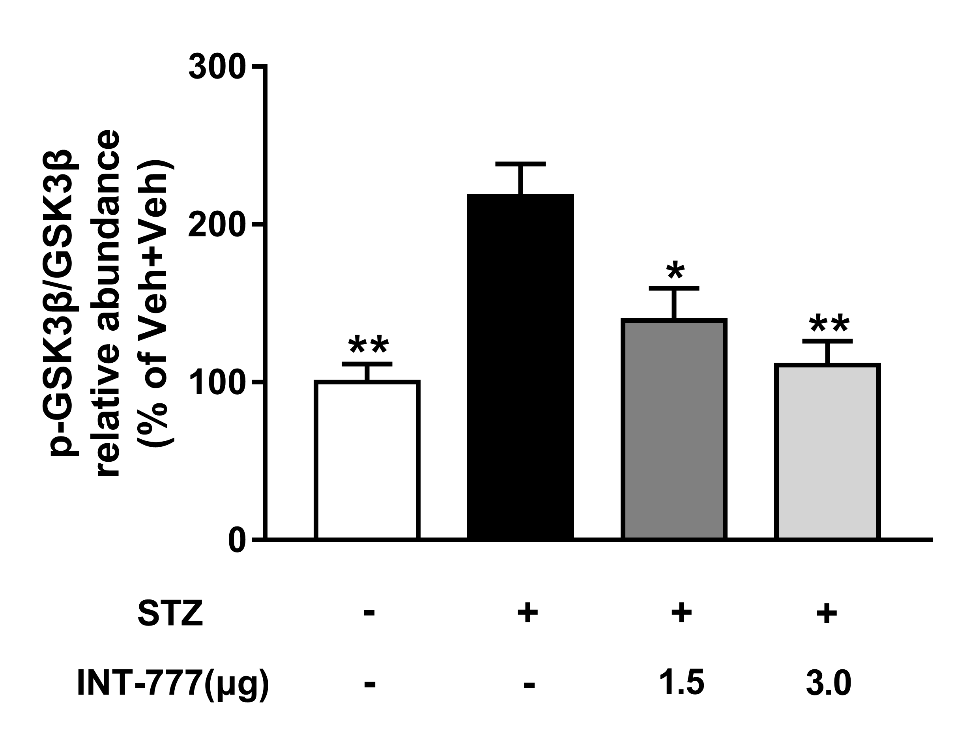


**Hippocampus**


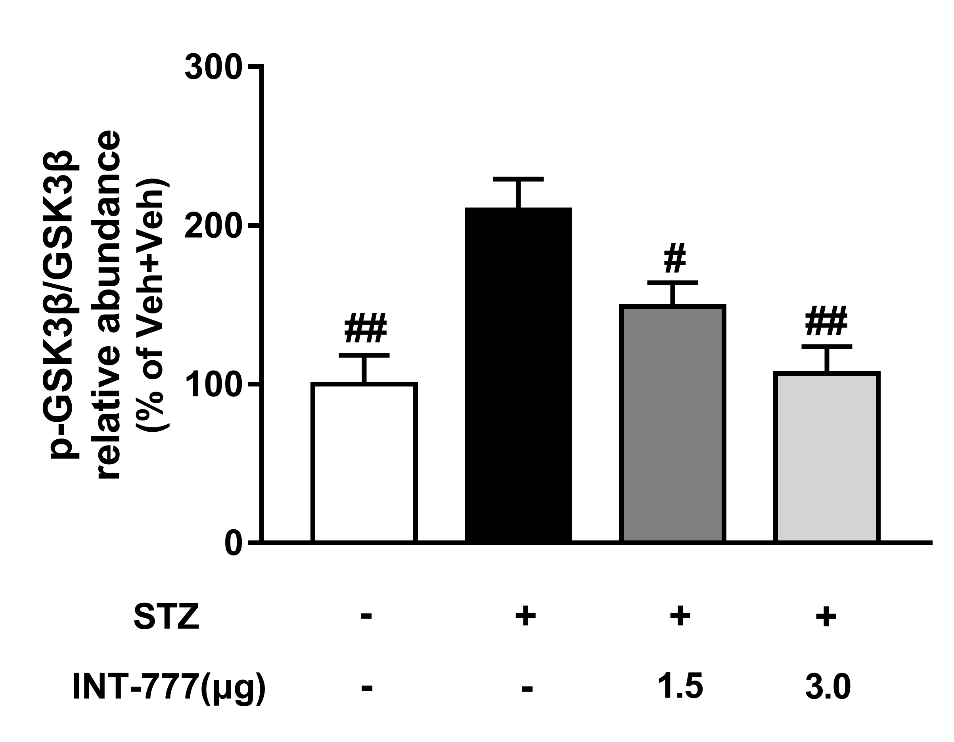


**Frontal cortex**

**Fig. S2** INT-777 alleviates STZ-induced activation of GSK3β in the hippocampus and frontal cortex. (**A**) GSK3β phosphorylation of the hippocampus and frontal cortex was detected by western blot. β-actin was used as a loading control. (**B**) Quantification of GSK3β activation was expressed as the ratio of p-GSK3β/ GSK3β. Values shown are expressed as mean ± S.E.M; n = 3 mice/group. ^*^*P* < 0.05, ^**^*P* < 0.01 vs. the hippocampus of STZ + Veh group; ^#^*P* < 0.05, ^##^*P* < 0.01 vs. the frontal cortex of STZ + Veh group.

**A**


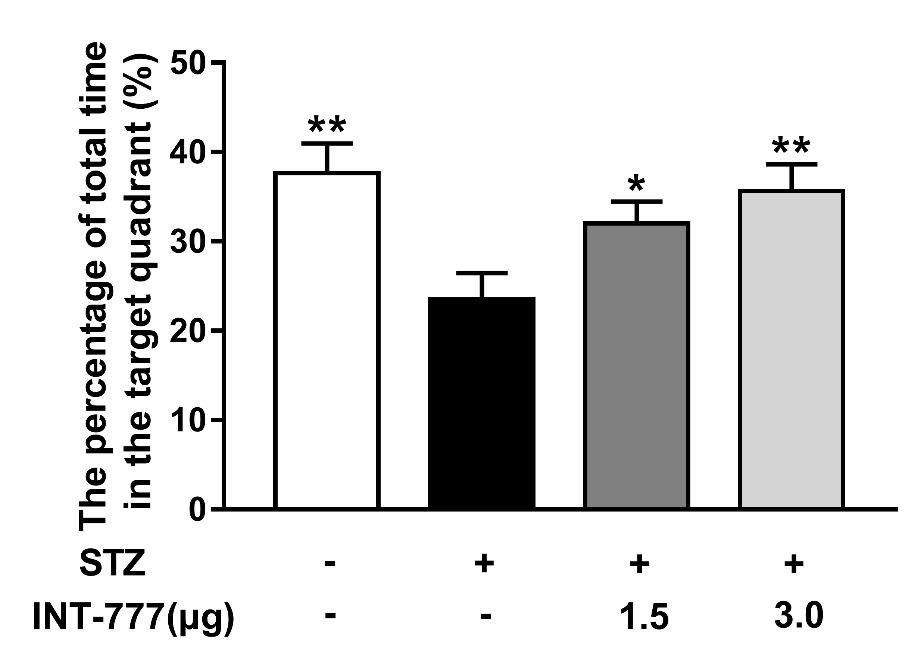

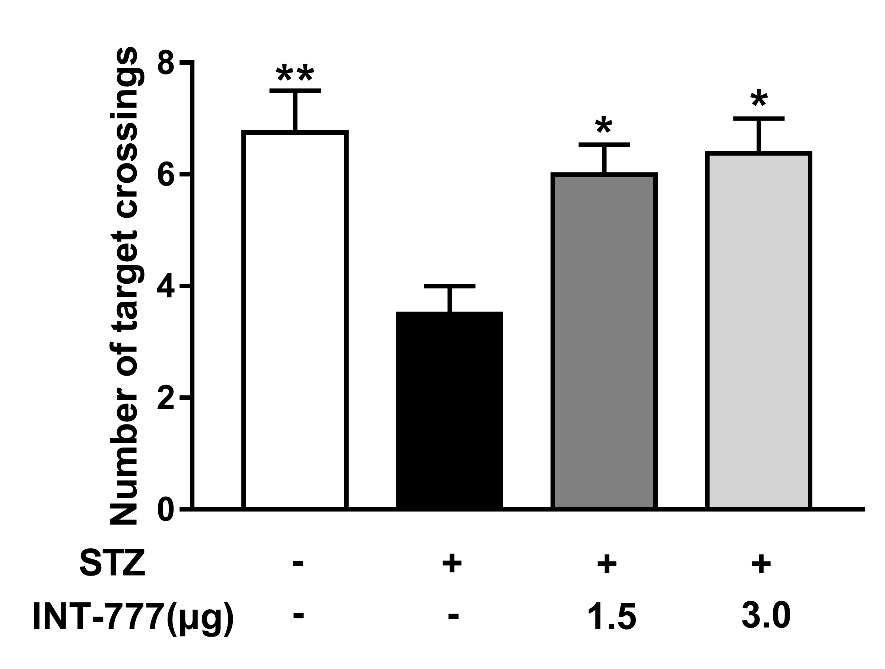


**B**


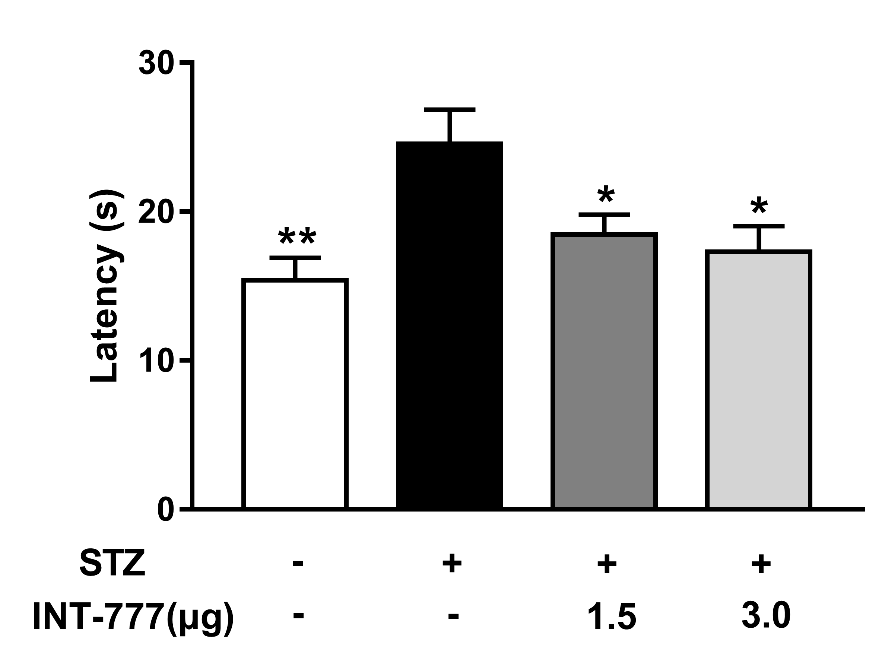


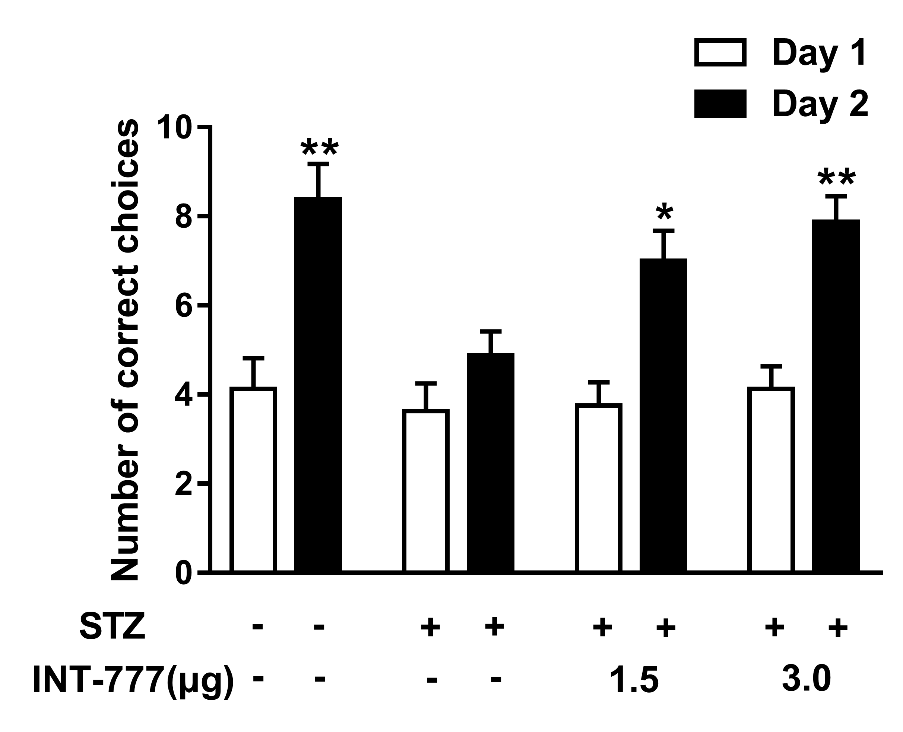


**Fig. S3** INT-777 ameliorates STZ-induced cognitive impairment in C57BL/6 mice before electrophysiological recordings in brain slices. (**A**) The percentage of time spent in the target quadrant in MWM test. (**B**) The numbers of platform location crossings during the probe trial in MWM test. (**C**) The number of correct choices on days 1-2 in Y-maze test. (**D**) The latency to enter the shock-free compartment on day 2 in Y-maze test. Values shown are expressed as mean ± S.E.M; n = 8 mice/group. ^*^*P* < 0.05, ^**^*P* < 0.01 vs. STZ + Veh group.

**A**

**B**


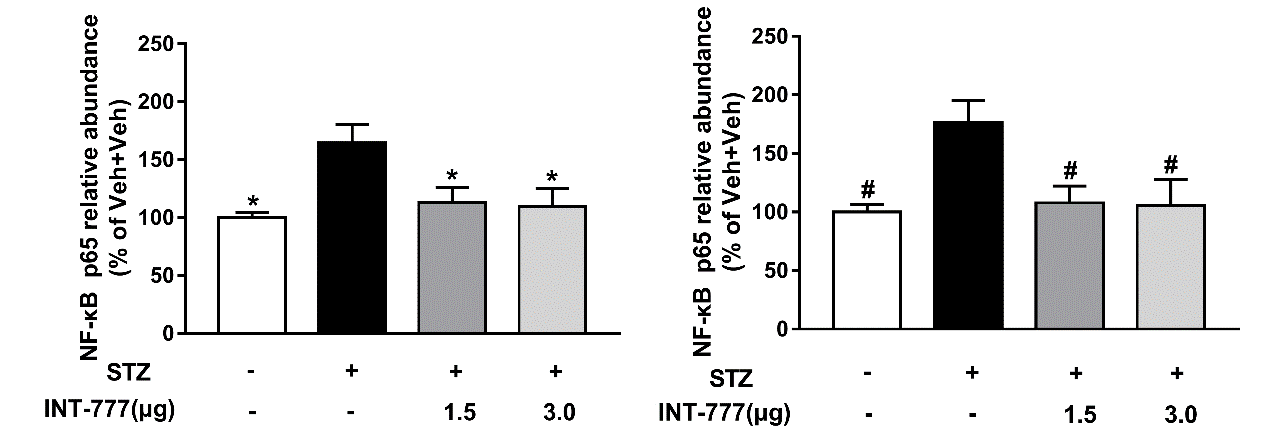


**Hippocampus**

**Frontal cortex**


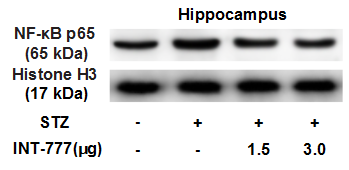

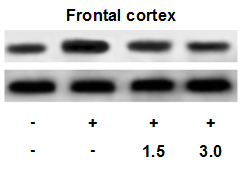


**Fig. S4** INT-777 suppresses STZ-activated NF-κB signaling in the hippocampus and frontal cortex. (**A**) NF-κB p65 protein was detected by western blot while Histone H3 was used as a loading control. (**B**) Quantification of NF-κB p65 was expressed as the ratio (in percentage) of Veh + Veh group. Values shown are expressed as mean ± S.E.M; n = 3 mice/group. ^*^*P* < 0.05 vs. the hippocampus of STZ + Veh group; ^#^*P* < 0.05 vs. the frontal cortex of STZ + Veh group.


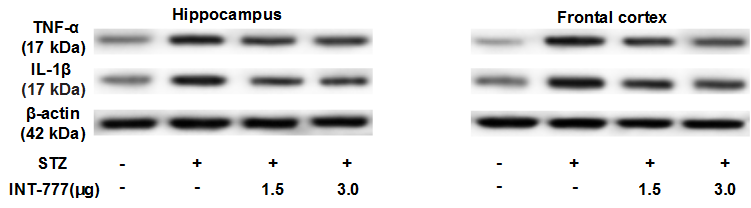


**A**

**B**


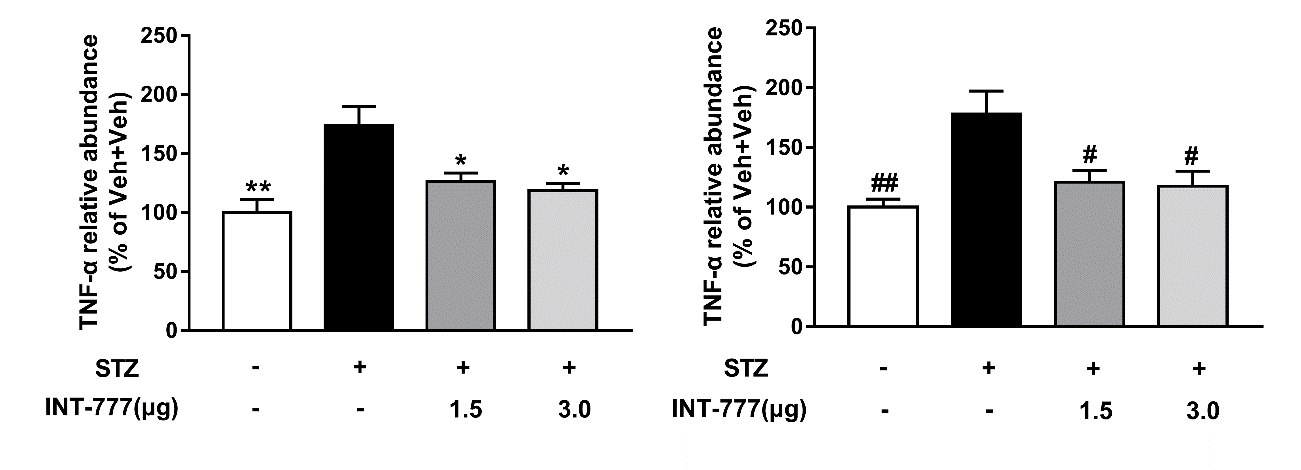


**Hippocampus**

**Frontal cortex**

**C**


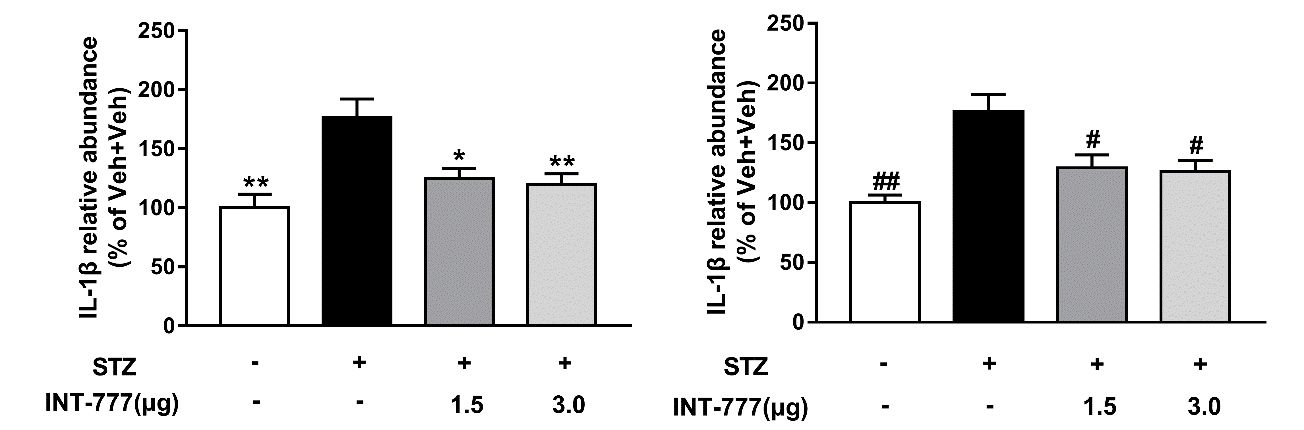


**Hippocampus**

**Frontal cortex**

**Fig. S5** INT-777 decreases the production of proinflammatory cytokines induced by STZ in mice. (**A**) Representative immunoblots of TNF-α and IL-1β in the hippocampus and frontal cortex were shown; β-actin was used as a loading control. (**B** and **C**) Quantifications of TNF-α (**B**), and IL-1β (**C**) were expressed as the ratio (in percentage) of Veh + Veh group. Values shown are expressed as mean ± S.E.M; n = 3 mice/group. ^*^*P* < 0.05, ^**^*P* < 0.01 vs. the hippocampus of STZ + Veh group; ^#^*P* < 0.05, ^##^*P* < 0.01 vs. the frontal cortex of STZ + Veh group.


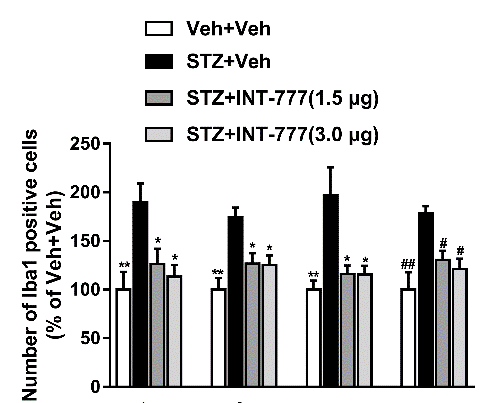

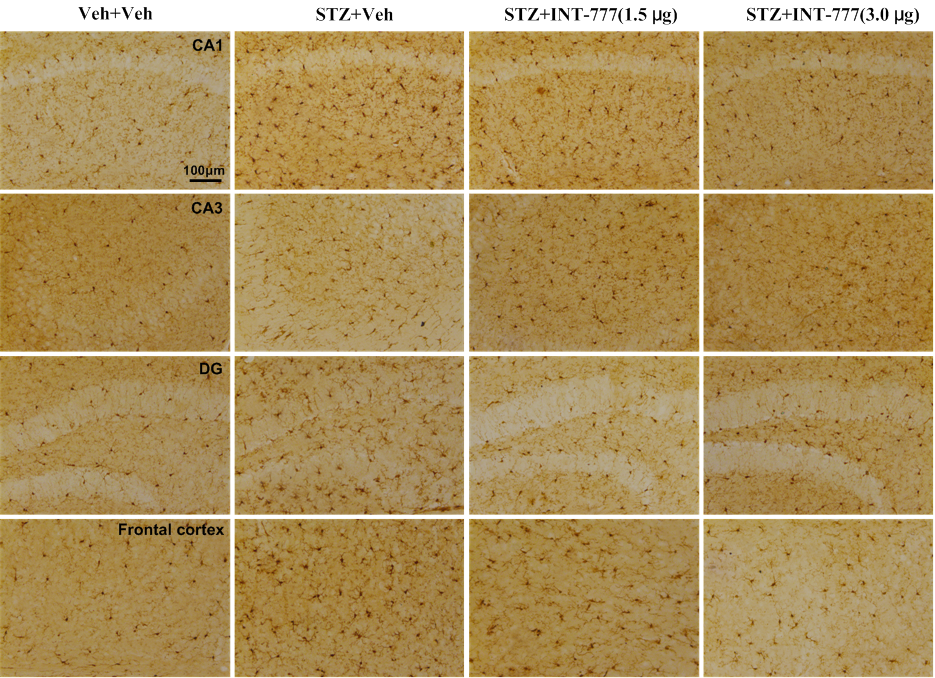


**A**

**B**

**CA1**

**CA3**

**DG**

**FC**

**Fig. S6** INT-777 suppresses STZ-induced microglia activation in the hippocampus and frontal cortex. (**A**) Representative images of Iba1-labelled activated microglia in the hippocampal CA1, CA3 and DG regions, and frontal cortex were shown by IHC. Scale bar: 100 μm. (**B**) The number of Iba1-positive cells was normalized in the corresponding same area, as the ratio (in percentage) of the Veh + Veh are shown. Values shown are expressed as mean ± S.E.M; n = 3 mice/group, four sections per mouse. ^*^*P* < 0.05, ^**^*P* < 0.01 vs. the hippocampus of STZ + Veh group; ^#^*P* < 0.05, ^##^*P* < 0.01 vs. the frontal cortex of STZ + Veh group.
